# Nanocellulose-based porous materials: Regulation and pathway to commercialization in regenerative medicine

**DOI:** 10.1016/j.bioactmat.2023.06.020

**Published:** 2023-07-12

**Authors:** Filipe V. Ferreira, Alana G. Souza, Rubina Ajdary, Lucas P. de Souza, João H. Lopes, Daniel S. Correa, Gilberto Siqueira, Hernane S. Barud, Derval dos S. Rosa, Luiz H.C. Mattoso, Orlando J. Rojas

**Affiliations:** a Nanotechnology National Laboratory for Agriculture (LNNA), Embrapa Instrumentation – Rua XV de Novembro, 1452, São Carlos, SP, 13560-979, Brazil; b Center for Engineering, Modeling, and Applied Social Sciences (CECS), Federal University of ABC (UFABC), Santo André, Brazil; c Department of Bioproducts and Biosystems, School of Chemical Engineering, Aalto University, P. O. Box 16300, Aalto, Espoo, FIN-00076, Finland; d College of Engineering and Physical Sciences, Aston Institute of Materials Research, Aston University, Birmingham, UK; e Department of Chemistry, Division of Fundamental Sciences (IEF), Technological Institute of Aeronautics (ITA), São Jose dos Campos, SP, Brazil; f Laboratory for Cellulose & Wood Materials, Empa - Swiss Federal Laboratories for Materials Science and Technology, Überlandstrasse 129, 8600, Dübendorf, Switzerland; g Biopolymers and Biomaterials Laboratory (BIOPOLMAT), University of Araraquara (UNIARA), Araraquara, 14801-340, São Paulo, Brazil; h Bioproducts Institute, Department of Chemical & Biological Engineering, Department of Chemistry and, Department of Wood Science, The University of British Columbia, 2360 East Mall, Vancouver, BC, V6T 1Z3, Canada

**Keywords:** Cellulose nanofibrils, Cellulose nanocrystals, Regenerative medicine, Biomaterial, Sustainable materials, Green nanomaterials

## Abstract

We review the recent progress that have led to the development of porous materials based on cellulose nanostructures found in plants and other resources. In light of the properties that emerge from the chemistry, shape and structural control, we discuss some of the most promising uses of a plant-based material, nanocellulose, in regenerative medicine. Following a brief discussion about the fundamental aspects of self-assembly of nanocellulose precursors, we review the key strategies needed for material synthesis and to adjust the architecture of the materials (using three-dimensional printing, freeze-casted porous materials, and electrospinning) according to their uses in tissue engineering, artificial organs, controlled drug delivery and wound healing systems, among others. For this purpose, we map the structure–property–function relationships of nanocellulose-based porous materials and examine the course of actions that are required to translate innovation from the laboratory to industry. Such efforts require attention to regulatory aspects and market pull. Finally, the key challenges and opportunities in this nascent field are critically reviewed.

## Highlights

• Critical review of cellulose-based porous nanostructures in regenerative medicine.
• Key concepts for engineering porous materials are presented.
• Function-structure-property relationships at micro- and nano-scale are emphasized.
• The commercialization pathway and regulatory challenges are highlighted.

## Graphical abstract

**Figure:**
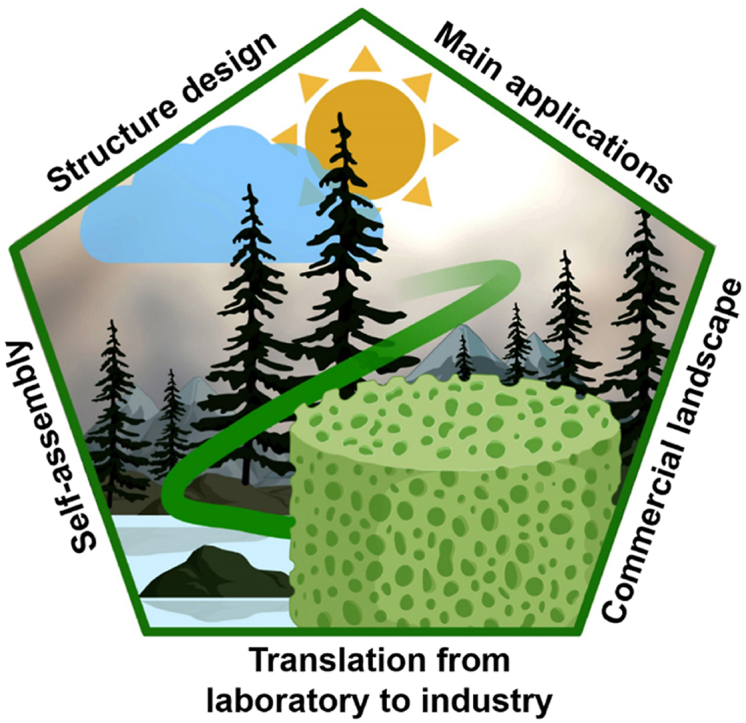

## 1 Introduction

Polymeric materials are present in our daily lives in applications ranging from household products to airplane components [[1], [2], [3]]. A significant number of these polymers is derived from fossil hydrocarbons [4], which may be chemically unsafe in some contexts, for instance, either because they are generally harmful to wildlife and threatening aquatic ecosystems [5] or because the unintended release of chemicals used in their production, use, and disposal [6]. Moreover, because of their recalcitrant nature, low recycling rates, and poor waste management, nonbiodegradable materials have become a major concern [7,8]. Geyer et al. [9] estimated that approximately 8300 million metric tons of polymers were produced in 2017, and most of them were accumulated in landfills. If this trend continues, the adverse environmental problems will continue to increase by an order of magnitude by 2025 [10]. This global situation is troubling and has been the focus of the global policies [[11], [12], [13]]. In many actions, interdisciplinary research has shed light on bio-based and renewable resources to answer the dependence on fossil resources to prepare materials that find use in our daily lives [[14], [15], [16], [17], [18], [19]]. Recent breakthroughs concerning cellulose-based materials are increasingly relevant in these efforts, and it is anticipated that they may replace a fraction of the market of petroleum-based polymers [[20], [21], [22], [23], [24], [25], [26], [27]].

Cellulose is virtually an inexhaustible natural polymer responsible for the structural functions in plants. Hierarchically, the microfibrils of cellulose align in tightly packed assemblies held together by hydrogen bonds and van der Waal forces [[28], [29], [30]]. Deconstruction of such structure by mechanical, chemical, or combination of mechanical and chemical, or enzymatic processes results in fibrillated celluloses, cellulose nanofibrils (CNF) or cellulose nanocrystals (CNC) – collectively referred to as nanocelluloses [[31], [32], [33], [34]]. CNF and CNC are versatile materials for manufacturing multifunctional micro- and nano-scale lightweight and renewable building blocks useful for many applications [[35], [36], [37], [38]]. Such ability emerges from their unique assembly into hierarchical and multiphase systems, as summarized elsewhere [[39], [40], [41]]. However, some aspects, including the guidelines for the adoption of CNF and CNC to achieve synergistic integration into porous materials have not been fully understood. Moreover, the critical actions (*i.e*., regulatory aspects and market pull) that are required to translate innovation from the lab scales to industry were not considered in other review papers. Herein, we introduce recent advances in the construction of micro- and nano-scale porous structures based on CNF and CNC. We use the available knowledge on the assembly process to understand the design principles that take advantage of nanocellulose’s self-assembly features. We begin by briefly discussing the fundamental principles of nanocellulose chemistry and self-assembly. We then summarize the methods to integrate nanocellulose-based hierarchically porous structures, highlighting fundamental concepts and synthesis strategies. In particular, the critical features of surface interaction and wettability of CNF and CNC are discussed to modulate structure–properties relationship. This is accomplished from the design principles to the final performance in innovative applications in regenerative medicine. We conclude with an in-depth account on the future direction and opportunities for designing and fabricating such nanocellulose-based porous materials towards translation from laboratory to industry. This highly cross-disciplinary contribution will serve the scientific community, including those starting discoveries in the area of materials chemistry (*e.g.*, self-assembly, cellulosic nanostructures, polymer nanocomposites, *etc*.), as well as practitioners interested in the translation, from innovation to commercialization. In sum, this review adds to current understanding of the nanocellulose-based porous materials engineering, paving the route for tailored design for versatile advanced applications, helping to overcome a major challenge that exists in the preparation of such materials, namely, achieving the control of design–performance relationship, as well as their commercialization.

## 2 Nanocellulose isolation, chemistry, and self-assembly

Nanocellulose isolation [[42], [43], [44]], chemistry [[45], [46], [47]], and self-assembly [44,48,49] have been discussed in depth in other review papers published in leading journals. Thus, these issues were not discussed in depth here. Briefly, nanocellulose isolation refers to the process of extracting and obtaining nanoscale cellulose structures from cellulose-rich sources [50]. Such process is a critical step in harnessing the potential of nanocellulose for a wide range of applications, and depending on the source, method, and chemicals used, the resulting nanocellulose can vary in structure and properties (*i.e.*, crystallinity, morphology, aspect ratio, and surface chemistry) [51]. Nowadays, different methods have been used for isolating such materials [[52], [53], [54]], but the mechanical process and acid hydrolysis occupies an important position, representing the foremost route toward CNF and CNC isolation [[55], [56], [57]].

The chemistry on the surface of cellulose plays an important role in determining its properties and interactions with other materials. The abundant hydroxyl groups (–OH) enable modification of nanocellulose surfaces by catalytic [58], polymerizable [59], and ionic [60] groups, which are desirable to expand the application spectrum [61]. Surface functionalization of nanocellulose can also be achieved through coating processes, where a thin layer of another material is deposited onto the cellulose surface, imparting new properties such as barrier properties, adhesion, or optical characteristics [62,63]. By understanding and manipulating the surface chemistry, researchers can expand the potential of nanocellulose as a renewable and versatile material.

**Fig. 1:**
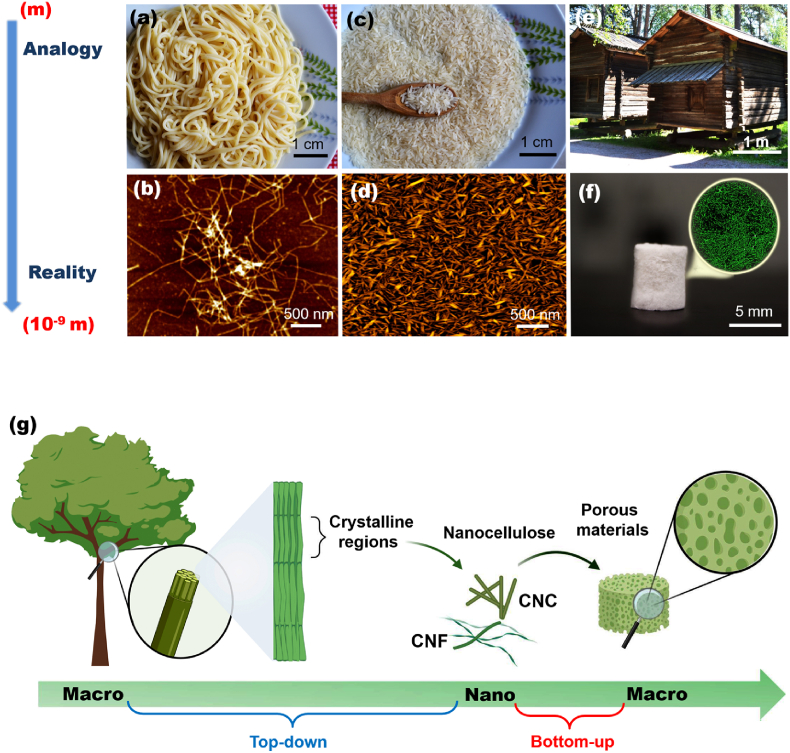
(a) Spaghetti and (c) rice grains are visualized as analogues* to (b) CNF and (d) CNC that can be used in the construction of hierarchical structures, represented by a (e) traditional Finnish wooden house or (f) micro- and nanostructured cryogels. (g) Scheme to introduce the (a) top-down and bottom-up approaches to prepare nanocellulose-based porous functional materials. *Analogy for better understanding, differences must be taken into account, *e.g*., the flexibility of CNF is completely different from noodles.

In contrast to CNF, which are plant nanofibers with a structure with high axial aspect ratio, measuring 5–500 nm in diameter and lengths of up to a few micrometers (Fig. 1a and b), CNC consists of a rod-like morphology (Fig. 1c and d), with lengths ranging from 100 to 1000 nm and diameter of 5–20 nm [64]. Other nanocelluloses properties include their crystallinity (especially for CNC) [65], versatility for surface modification [66,67], good mechanical properties (estimated elastic modulus and tensile strength in the range of 105–168 and 0.3–22 GPa, respectively) [68,69], and self-assembly ability [70]. This latter property has been extensively investigated for CNC [[71], [72], [73]]. Such self-assembly is driven by various intermolecular forces (*e.g*., hydrogen bonding and van der Waals interactions), which promote the alignment of nanocellulose [74]. This hierarchical organization process can be influenced by factors such as concentration, pH, temperature, and the presence of additives or surfactants [48,75]. By controlling the process, materials with desired characteristics (*e.g*., mechanical strength, optical properties, and responsiveness to environmental stimuli) can be can be achieved for different applications in the fields of packaging, tissue engineering, drug delivery, and sensors [44,49,76]. Other (bio)molecules (*e.g.*, proteins [77]) and inorganic substances (*e.g.*, transition metal oxide [78]) may also be used in the assembling processes [79] for fabricating functional materials [80].

Due to their outstanding properties, cellulose-based structures have been engineered, and promising results for different applications have been proposed [[81], [82], [83], [84]]. In particular, CNF and CNC have received a great deal of attention as precursors of porous structures (Fig. 1f). Two different approaches are used for the synthesis and production of cellulose materials, *i.e*., top-down and bottom-up methods [85]. Top-down is mainly related to the deconstruction of the larger cellulose structures (*e.g*., wood pulp or cellulose fibers) through physical or chemical treatment (*e.g*., mechanical disintegration, high-pressure homogenization, or chemical hydrolysis) to obtain nanoscale cellulose structures (*i.e*., CNC and CNF) [86]. On the other hand, bottom-up involves the assembly or synthesis of cellulose-based materials from such nanoscale cellulose structures [87]. In this approach, the materials are formed by self-assembly or controlled synthesis [88]. Overall, the choice of these approaches depends on the specific purpose and desired application [89]. Herein, we focus on bottom-up approach (Fig. 1g), where the extracted nanocellulose is assembled into porous functional materials.

## 3 Hierarchically porous materials - synthesis strategies and structure design

Nanocellulose-based porous structures are an emerging materials class and the integration with strategies used for their production enable designs with remarkable functions. In particular, we summarize the latest advances aimed at developing mechanical strength, porosity and orientation for uses in regenerative medicine. Herein, we highlight the fundamental concepts related to the strategies to achieve well-controlled nanocellulose-based porous materials, including three-dimensional (3D) printing, freeze-casted porous materials, and electrospinning.

### 3.1 Three-dimensional (3D) printing

**Fig. 2:**
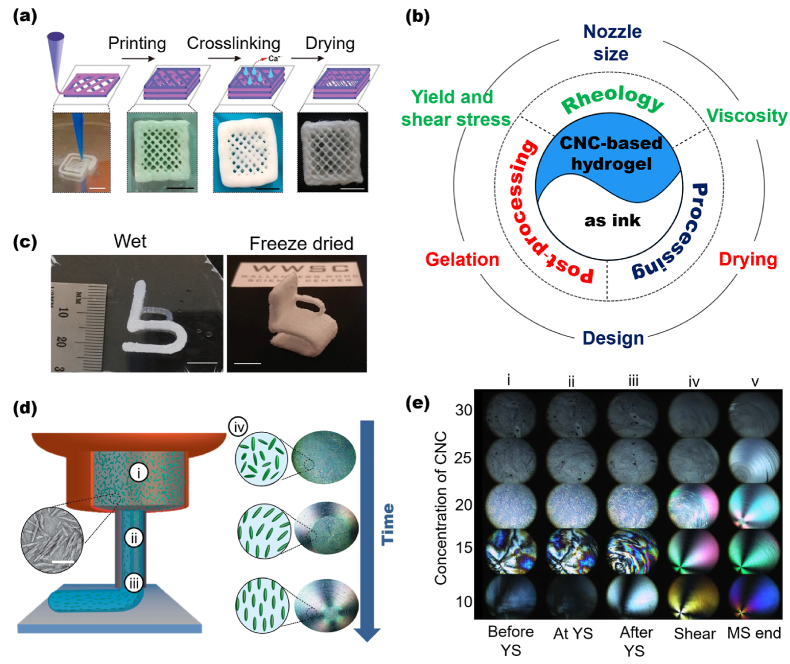
(a) 3D printed CNF-based cubic scaffolds (scale bar is 5 mm). (b) Main parameters that must be taken into account when preparing a material by 3D printing. Green: rheological properties (*i.e.*, viscosity and shear stress); blue: processing (*i.e.*, nozzle size and fabrication time); red: post‐processing (*i.e.*, gelation and drying). (c) 3D printed small chair based on nanocellulose showing the high level of detail after freeze-drying. The scale bar corresponds to a 10 mm. (d) Scheme showing that 3D printing nozzles combined with shear and extensional flow can be used to tune the orientation of CNC. (d_i_) CNC randomly organized; (d_ii_) Aligned CNC due to maximized shear stresses through the nozzle; (d_iii_) Final oriented extruded segment. (d_iv_) Alignment of CNC observed by polarized light imaging. (e) Alignment of a CNC as a function of concentrations and shear-stress. (e_i_) Before, (e_ii_) at, and (e_iii-iv_) after yield stress (YS); (e_v_) at the end of the measurement. (a) Adapted from Ref. [95] with permission of Copyright 2019 American Chemical Society. (b) Inspired from Ref. [96]. (c) Adapted from Ref. [97] with permission of Copyright 2016 Wiley-VCH. (d–e) Adapted from Ref. [98] with permission of Copyright 2018 American Chemical Society.

3D printing has been adopted as one of the main approaches for fabricating porous materials because it allows the preparation of complex structures that cannot be achieved with conventional manufacturing approaches [90,91]. The engineered porous materials are developed using computer-assisted technology and, generally, the 3D printing methods are primarily classified as extrusion, inkjet, laser-assisted, and UV-assisted printing [92]. Extrusion occupies an essential position among these methods due to its high throughput and compatibility with different inks [93]. The method is based on the extrusion of hydrogel inks which are then solidified as post-processing removes the solvent. A wide variety of natural and synthetic polymers can be employed as inks in 3D printing [94], including nanocellulose-based hydrogels. In this case, the nanocelluloses are seamlessly intertwined in a viscous hydrogel with both structural complexity and mechanical behavior modulated by the interaction of the cellulose chains, especially through crosslinking process (Fig. 2a) [95]. Adjusting the physicochemical parameters that affect nanocellulose-based hydrogel properties is one of the main strategies for preparing porous structures with shape fidelity and mechanical stability. Here, we review recent 3D printing innovations based on nanocellulose. Specific requirements that determine the printability of the hydrogel to achieve reliable fabrication of 3D constructs are illustrated in Fig. 2b and discussed next in more detail.

The conversion of nanocellulose-based hydrogels into a dry 3D structure with good shape fidelity is one of the main challenges in 3D printing because cellulose chains can collapse upon drying, changing the printed form [97]. The rheological properties (*i.e.*, viscosity, yield and shear stress), processing (*i.e.*, nozzle size and design), and post‐processing (*i.e.*, gelation and drying) are some of the main aspects to consider in the preparation of nanocellulose-based aerogels or porous materials by 3D printing [96].

Several 3D printing technologies can be used to manufacture synthetic heterogeneous nanocellulose-based porous materials such as stereolithography (SLA), digital projection lithography (DLP) and direct ink writing (DIW) [99,100]. Nanocellulose-based materials produced by DIW, an extrusion-based 3D printing approach, can be utilized in a wide range of materials of different sizes and geometries. The main challenges related to this technique relates to the development of viscoelastic inks that are ready for extrusion, and yet form self-supporting features after exiting the nozzle [101]. Thus, a printable ink should have shear thinning behavior; G′ >G″ with G′ of the order of few kPa and apparent yield stress of few hundred Pa [102].

The viscosity of inks can affect the printing fidelity and dictates its gelation, *i.e.*, how quickly it needs to solidify [96]. Low zero-shear viscosity gives hydrogel inks a poor shape fidelity when printing and a slow gelling, leading to the collapse of the printed structure [103]. High viscosity inks may take less time to gelation, but very fast gelation can clog the needle during printing. In general, the hydrogel needs to be viscous enough to flow in the printer, becoming solid-like shortly after extrusion [104,105]. In this sense, the outstanding shear thinning properties of nanocellulose hydrogels allows flow from the needle when the stresses are present, but when deposited, a 3D structure made with relaxed hydrogel is formed [98]. Ink formulation must also be taken into account. For instance, a hydrogel with 5 wt% of CNC (low viscosity) and a hydrogel with 20 wt% CNC can polymerize both quickly (or in a very similar time) if a monomer and a photo-initiator were used [106].

Regarding cellulose chain collapse during gelation, it can also be controlled using different drying processes (*e.g.*, air drying, solvent exchange before drying, and freeze-drying) [97,107] and/or through crosslinking [95,108]. In this sense, the high density of nanocellulose surface charges can tailor the gelation due to crosslinking [[109], [110], [111]]. Therefore, printed 3D structures with controlled architectures are preserved upon solidification (Fig. 2c). The combination of surface charged-nanocellulose and alginate can promote the crosslinking process, preparing post‐processed material with shape fidelity and sufficient mechanical stability [112]. The viscosity of the hydrogel can be conveniently controlled by nanocellulose concentration. Moreover, considering the relationship viscosity–gelation on fabrication time of the printed material, viscosity is also a key mechanism to control fabrication time [113].

Efforts to alter the hydrogel rheology – consequently, the mechanical behavior of the final material – have combined the nanocellulose with other polymers. Ajdary et al. [114] added CNF into poly(glycerol sebacate) (PGS) and polypyrrole (PPy) as an active component to alter the elasticity and flexibility of the printed material facilitating direct ink writing. Functional components such as carbon nanotubes [115,116], graphene [117], graphene oxide [118], and magnetic nanoparticles [119] have been added to the inks to open the development of 3D printed materials for different applications.

The nozzle size is an additional factor that influences both drop volume and shear stress, and consequently, the printed material properties [120]. Hausmann et al. [98] showed that 3D printing nozzles combined with shear and extensional flow could be used as effective means to tune the orientation of CNC, therefore, affecting the manufactured material’s microstructure and properties. The authors observed that the CNC might be aligned during extrusion due to the shear stress imposed on the suspension (Fig. 2d). During printing, the nanocrystals randomly distributed in the cartridge are then aligned due to high shear stresses that exist through the nozzle, resulting in a final oriented extruded segment (Fig. 2di-iii). Such alignment of CNC can be observed by polarized light imaging (Fig. 2div), and it is a function of CNC concentrations and shear-stress (Fig. 2e). Gladman et al. [121] took advantage of the extensional and shear forces experienced during DIW to align CNF building blocks while printing. They demonstrated how to create bilayer architectures with programmable anisotropic swelling by controlling the printing parameters and CNF orientation within the filaments, opening opportunities to create soft materials for tissue engineering, biomedical devices and soft robotics.

### 3.2 Freeze-casted porous materials

Freeze casting is a versatile, cost-effective, and rapid method for fabricating well-controlled porous structures based on nanocelluloses. Freeze-casting is well-suited to produce porous materials by using, for example, nontoxic (and inexpensive) precursors, eliminating or reducing the usage of solvents, and decreasing production cost. Traditionally, the processing occurs by two main processes: controlled solidification of wet foam/gel and sublimation of the solvent (such as water) under reduced pressure.

(i) Controlled solidification of wet foam/gel: First, a multi-phase dispersion containing nanocellulose in a liquid phase (usually water) is considered. Other solvents, including ethanol, can also be used as pore-creating agents. However, intermediate steps involving solvent exchange may be required [122]. A critical step is to ensure the separation of nanocellulose by the liquid phase because these particles serve as building blocks (walls of the porous material) in the process [123]. In general, the separation step occurs when the interfacial free energies satisfy the following criterion (Equation (1)):

(1) $${\Delta \gamma }_{0}=\gamma_{ps}-\left(\gamma_{pl}+\gamma_{sl}\right)>0$$

where, $\gamma_{ps}$, $\gamma_{pl}$ and $\gamma_{sl}$ are the interfacial free energies associated with the particle/solid, particle/liquid, and solid/liquid interfaces, respectively [123].

**Fig. 3:**
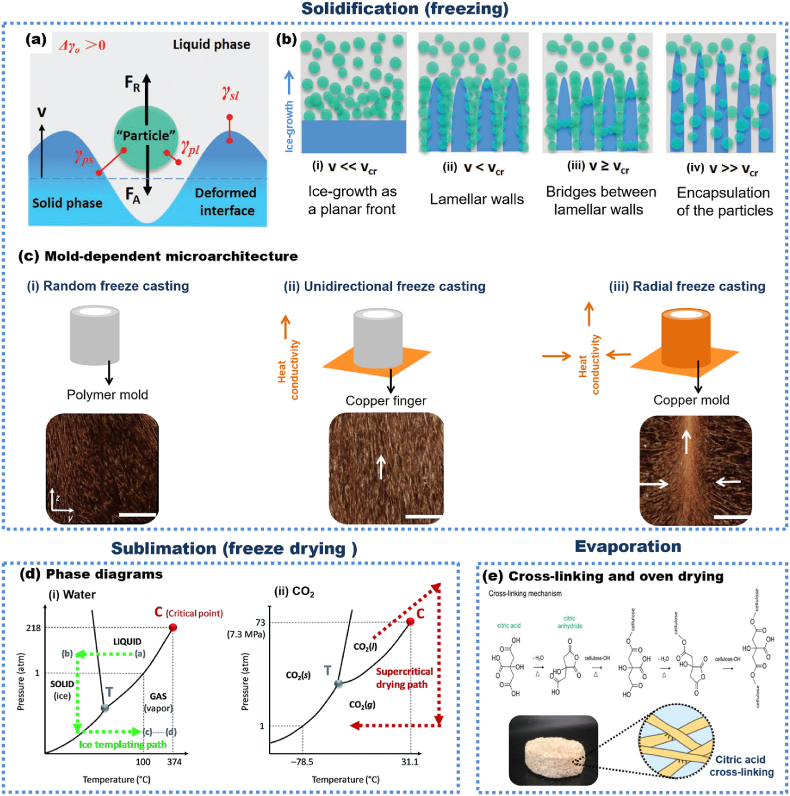
(a) Forces acting on the particles at the liquid-solid interface during particulate in the vicinity of a freezing front. (b) Effect of freezing front velocity (ν) and critical freezing front velocity $\left(\nu_{cr}\right)$ on final pore morphologies of freeze cast material. (c) Mold-dependent microarchitecture of CNF-based cryogel - polyethylene copper molds (top) used for freezing CNF suspensions. X-ray microtomography reconstructions (μCT; bottom) of the obtained porous material (scale bar = 5 mm). (d) Phase diagrams of (d_i_) water and (d_ii_) carbon dioxide (CO_2_). (e) Cross-linked cellulose to prepare porous materials by evaporation in the oven. (a–b) Adapted from Ref. [123] with permission of Copyright 2020 Wiley-VCH. (c) Adapted from Ref. [128] with the permission of Elsevier. (d) Reproduced from Ref. [129] with permission from Copyright 2017 Royal Society of Chemistry. (e) Adapted from Ref. [130] with permission of Copyright 2020 American Chemical Society.

Phase separation occurs through solvent solidification (freezing). Thermodynamically, repulsive (FR) and attractive (FA) forces act on the particles at the liquid/solid interface during particulate suspensions (Fig. 3a). The final pore morphology of freeze cast materials is significantly affected by the relationship between freezing front velocity (ν) and critical freezing front velocity $\left(\nu_{cr}\right)$, as illustrated in Fig. 3b and discussed by Shao et al. [123] Briefly, pores of freeze cast structures do not formed when freezing rates are very low (${\nu \ll \nu }_{cr}$; Fig. 3bi). On the other hand, lamellar walls are obtained if ${\nu <\nu }_{cr}$ (Fig. 3bii). When ${\nu \geq \nu }_{cr}$ (Fig. 3biii), some nanocellulose can be entrapped by solid phase growth, yielding a different porosity scale than that observed otherwise. Finally, at very fast freezing rates (${\nu \gg \nu }_{cr}$; Fig. 3biv), the encapsulation of nanocellulose within the ice front can occur [123]. The freezing process govern these freezing front velocities from freeze casting (*i.e.*, in a freezer or liquid nitrogen) and the temperature gradient of mold material [124]. The columnar or lamellar morphologies can be achieved through unidirectional and radial freeze casting [125], whereas the size of the pores is scales with the solidification rate [126], given that fast freezing prevents the phases segregation [127]. Mariano et al. [128] tailored the porous orientation (Fig. 3c, white arrows) through heat conductivity of the mold material (*i.e.,* freezing on copper is faster than polyethylene), which affect the ice-crystals growth direction, and the obtained porous structure.

Importantly, along with pore tuning in terms of size and shape by freezing velocity, other variables offer opportunities to tailor the structure of freeze-cast materials. The generation of gas bubbles can achieve slight control of the size and shape of the formed pores through high-intensity stirring or air sparging [131]. In this sense, the porosity and orientation usually increase as bubbles and/or bubble orientation increase. The type of nanocellulose used (CNC, CNF) is critically important. Munier et al. [132] showed that unidirectional freezing of CNC dispersions leads to porous materials with the high alignment in the freezing direction. However, compared to the CNF (0.08 wt %), the concentration of particle concentrations should be increased using CNC (above 0.2 wt %), leading to 3D structures with high orientation. This behavior occurs because CNF forms a so-called “3D maze” of entangled nanofibrils at a very low solids content [133]. Such structure is especially beneficial where directional conduction is needed, *e.g.*, for sunlight evaporators [134] and energy harvesting [135].

(ii) Solvent Sublimation: The solidified solvent (suspension media) is removed by freeze-drying or supercritical drying (Fig. 3d) [129,136]. The process involves the removal of solidified suspension by lowering the pressure. No parameters have been reported to play a significant role in the development of micro- and macrostructures of the porous materials. In recent years, evaporation in ovens has been shown as alternative to replace freezing casting (solidification and sublimation). The mechanism of swelling and collapse for these porous materials involve unique cellulose-cellulose interactions [129]. The interaction forces between nanocellulose and solvent are essential to ensure the formation of distinct phases and to template removal by evaporation. During drying, the energetic cost of holding polymer chains separated should not be as significant as the energetic gain of the interaction due to hydrogen bonding between cellulose chains. Functional groups attached to the nanocellulose surface [137] and/or crosslinkers [138] can increase the energy cost for a collapsed conformation, allowing the preparation of porous materials based on nanocellulose with no need for freeze-casting. For instance, Ferreira et al. [130] prepared lignocellulosic foams by oven-drying sugarcane bagasse fibers cross-linked in the presence of citric acid (Fig. 3e).

### 3.3 Electrospinning

**Fig. 4:**
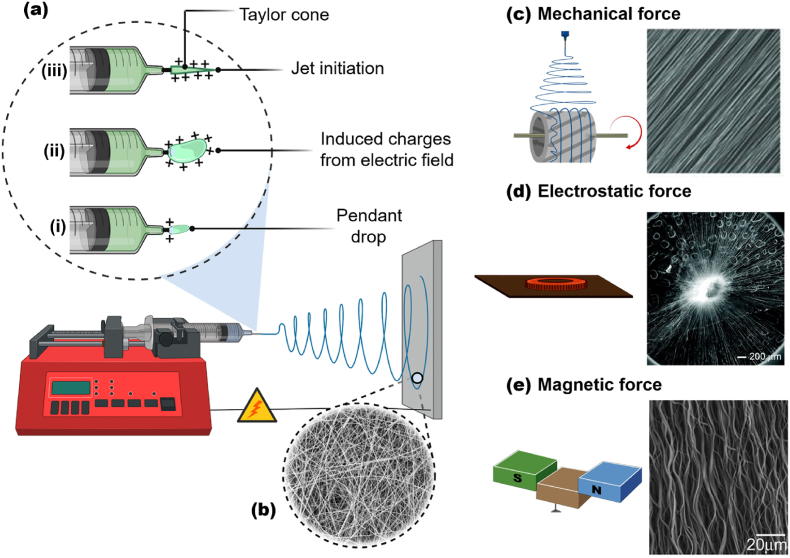
(a) Traditional electrospinning apparatus containing a high-voltage power supply, a syringe with spinneret, a syringe pump, and a grounded collector. Schematic illustration of electrospun fibers preparation: (a_i_) formation of surface charged droplets (a_ii_) extrusion of the solution from the syringe generating a tiny conical droplet. (a_iii_) “Cone-jet” regime where a fine jet initiates from the cone. (b) SEM images of electrospun fibers randomly oriented. Electrospinning set-ups, such as (c) mechanical, (d) electrostatic, and (e) magnetic forces to adjust pore structure and fiber orientation. The right column shows the aligned nanofibers prepared by different electrospinning set-ups. (a–b) Schematic diagram inspired from Ref. [140]. (c–e) Schematic diagram inspired from Ref. [149]. (c; right column) Adapted from Ref. [149] with permission of Copyright 2011 Wiley-VCH. (d; right column) Adapted from Ref. [145] with permission of Copyright 2010, American Chemical Society. (e; right column) Adapted from Ref. [147] with permission of Copyright 2010 Wiley-VCH.

Electrospinning is a versatile, simple, and useful technique that utilizes the electrostatic repulsion between surface charges of a polymer solution jet to fabricate micro- and nanofibers [139]. A generic electrospinning apparatus comprises a high-voltage power supply, a syringe, a syringe pump, and a grounded collector (Fig. 4a). Initially, surface-charged droplets (Fig. 4ai) are created by applying an electrical potential provided by a high-voltage power supply [140]. Then, the polymer solution is extruded from the syringe spinneret, generating a small conical droplet (Fig. 4aii), a Taylor cone [141]. Immediately, the droplet enters the “cone-jet” regime (Fig. 4aiii), and then the “whipping instability” regime, where the diameter of the jet is decreased over time while the solvent evaporates [142]. The final material is usually collected as a nonwoven mat composed of fibers with diameter from micrometers down to tens of nanometers (Fig. 4b). Pore structure and, especially, fiber orientation can be controlled by adjusting the experimental conditions, using rotating mandrel (Fig. 4c) [143,144], metallic materials (Fig. 4d) [145,146], and permanent magnets (Fig. 4e) [[147], [148], [149], [150]].

For electrospinning, and from the macromolecular point of view, nanocellulose should be dispersed in water-soluble, high molecular weight polymers. However, the processing flexibility enables the preparation of nanocellulose-based fibers using hydrophobic polymers. In such method, an additional step (*e.g.*, saponification with NaOH/ethanol and/or freeze-drying) might be needed, as well as some solvent can be required, depending on the polymer used [151,152]. The amount of nanocellulose is intimately linked to the material’s microstructure (*e.g.*, diameter, thermal and mechanical properties of the electrospun fibers). Shi et al. [153] prepared electrospun nanofibers of PLA reinforced with CNC from a solvent mixture consisting of *N,N*′-dimethylformamide, and chloroform at room temperature. The authors observed that the addition of CNCs up to 5 wt% improved the *in vitro* degradation, tensile stress and young’s modulus, and heat resistance of CNC-based electrospun fibers while decreased their average diameter. In this sense, many authors have studied how the addition of nanocellulose affects the overall properties of electrospun nanofibers. For instance, CNC affects the viscosity and electrical conductivity of the CNC-polymer solution. In particular, CNC can increase the viscosity of the solution, probably due to the formed network structure of within the polymer solution [[153], [154], [155]]. In contrast, the negative charges of CNC produced by sulfuric acid hydrolysis can decrease the electrical conductivity of the polymer solution [156].

Regarding the mechanical properties of nanocellulose-based electrospun fibers, in general, the high extensibility of the fibers (compared to ceramics-based counterparts) is unfavorable to maintain the shape and pore size but particularly useful to provide mechanical resistance to the fiber mat. These properties can be controlled by factors including individual fibers composition (*e.g.*, polymer matrix), mechanical percolation, and nanocellulose interfacial adhesion. Improved mechanical performance can be achieved by direct links between nanocellulose and the polymer due to percolation threshold and surface interaction [157]. To support high loads on the fiber’s long axis, the preparation of aligned fibers is another alternative [158]. This approach is powerful if a biomedical application is envisioned because aligned fibers positively affect cell functions [145,159,160]. Specifically, anisotropy in topography and structure can be highly desired for tendon/ligament, neural, and cardiac tissue engineering [144,161,162], where the fibrils are aligned parallel and/or perpendicularly interwoven [149]. Yin et al. [161] studied the effects of nanotopography (aligned or random poly(l-lactide)-based nanofibers) on the differentiation of human tendon stem/progenitor cells (hTSPCs). A higher expression in hTSPCs growing in aligned nanofibers was observed. Zong et al. [163] prepared fine-textured electrospun scaffolds for heart tissue constructs and observed modulation of tissue structure as a function of chemistry and geometry of the scaffold surface.

Molecules or nanoparticles can also be added during electrospinning. However, it is essential to keep in mind that such “fillers” also affect the viscosity of the suspension and, consequently, the electrospun fibers' diameter. Nano–biotherapeutic emulsion formulated with both CNF and CNC and multifunctional active compounds, *e.g.,* vegetable oils with antibiotic and antioxidant properties [164] and polypeptide nanoparticles [165], have been used in electrospun nanofibers with synergistic properties, including drug loading and release [166].

### 3.4 Comparison of 3D bioprinting, freeze-casting, and electrospinning

We highlight the versatility of nanocellulose for the preparation of hierarchically porous materials. 3D bioprinting, freeze-casting, and electrospinning are compared in Table 1 considering manufacturing aspects.

**Tables 1:** 3D bioprinting, freeze-casting, and electrospinning in the context of manufacturing.

Method	Advantages	Disadvantages
3D printing	• Materials can be easily shaped in any desired form (customization). • Complex structures can be prepared. • Significant material waste reduction.	• Often multi-step solvent-based techniques are required. • Costly fabrication with energy demand. • Shape fidelity and mechanical stability are difficult to achieve with inks prepared with nanocellulose alone.
Freeze-casting	• Typically, solvents are not required. • Aligned porous structures can be obtained.	• Complex structures are challenging to achieve
Electro-spinning	• Aligned nanofibrous mats of varied composition and morphology can be achieved.	• Harsh solvents may be required • Complex structures cannot be achieved

3D printing has become a compelling and universal method to prepare sustainable and versatile functional porous material. Complex structures such as blood vessels and organs can be engineered. However, 3D printing is a multi-step solvent-based technique [167,168]. Moreover, the drawbacks of shape fidelity and there are risks of collapsing cellulose chains upon drying (during post-processing). On the other hand, freeze-casting has been used as an “easy and green route” to prepare well-controlled porous structures based on nanocellulose. In this method, solidification (freezing) is crucial for templating and fabricating various structures with tunable pore sizes. Further aligned porous structures can be obtainable through recently developed freeze-casting techniques, which facilitate the material functionality across multiple fields of application [123]. However, unlike 3D printing, high structural complexity, *e.g.,* tissue-like materials, is still challenging to engineer by freeze-casting nanocellulose-based materials. Electrospinning gained much attention as a processing route for aligned nanofibrous mats. However, although control of pore structure and fiber orientation can be achieved by this technique, it does not yield 3D structures and is challenging to scale-up. However, some recent work has demonstrated scalable ultrathin fibers in areas relevant to this review [169].

In sum, the presented approaches are suitable to develop porous materials based on nanocellulose with distinct porosity range, especially meso (2–50 nm) and macro (>50 nm) porous. These synthesis routes have the potential to be scale-up for industrial applications. However, scaling-up while maintaining precise and simultaneous control of the overall properties (*i.e.*, porosity over pore geometries, mechanical performance, chemical functionalities, etc.) is still challenging. For instance, small changes in mass and heat transfer, are difficult to control, affecting the structure and consequently the properties of the fabricated porous materials [170]. In contrast to bottom-up approaches, top-down synthesis is an alternative to prepare hierarchically porous materials, taking advantage of the natural hierarchical structure of cellulose-based materials [171]. This approach does not necessarily use nanocellulose, but it can decrease time demanding manufacturing costs and energy needs.

## 4 Applications of nanocellulose-based porous materials

**Fig. 5:**
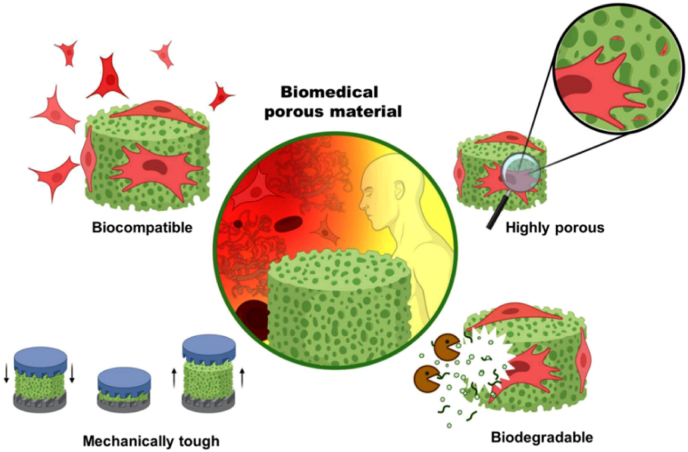
Main requirements for scaffolds applied in tissue engineering.

In recent years, CNF and CNC have been used to prepare highly porous biomaterials – or scaffolds– for regenerative medicine. Tissue engineering, in particular, is an important field that supports the formation of repair tissue that closely mimics native tissues [172]. Of the many scaffolds currently utilized for tissue engineering, those based on nanocellulose are quite promising [20,173,174]. In general, scaffolds should transduce changes in the physiological environment into therapeutic response [[175], [176], [177]]. For this purpose, biomedical materials should be biocompatible, highly porous, mechanically tough, bioactive, and biodegradable (Fig. 5) [173,178,179].

The biocompatibility of the scaffolds means minimal toxicity (*i.e.*, medical therapy without eliciting any undesirable local or systemic effects in the recipient) and favorable biological interactions with the surrounding tissue (*i.e.*, bioactivity). From the perspective of the tissue scaffold, efforts have been made to increase the bioactivity while maintaining the porous structure and tenability. Such efforts have led to a new generation of nanocellulose-based composites scaffolds and exciting prospects for application in clinical settings.

Porous materials are classified into three categories according to the International Union of Pure and Applied Chemistry - IUPAC [180]: microporous (<2 nm), mesoporous (2–50 nm), or macroporous (>50 nm). For biomedical applications, the porosity of scaffolds is critical to provide sufficient space for cell functions (*i.e.*, growth, migration, and proliferation). Moreover, damaged tissue repair is hindered due to the lack of scaffolds vascularization. Since 30–40 μm is the minimum porosity required for proper scaffold vascularization [181], larger pores (160–270 μm) facilitate vascularization and gas exchange and nutrient transport in the scaffold, hence macropores materials are more appropriate for this purpose. Unfortunately, increasing porosity to promote the suitable biomimetic microenvironment for cell functions and neovascularization might compromise the mechanical strength of the material [173]. Regarding compression, crosslinking can facilitate the preparation of porous materials with versatile deformation and shape recovery behavior.

The mechanical properties of scaffolds should mimic the properties of the surrounding environment [182]. In this context, low porosity imparts better scaffolds' mechanical stability and provides applications in hard tissues; meanwhile more porous (softer) materials are better suited for soft tissues. In all cases there is a need for balancing a suitable mechanical performance and facilitating the proper nutrient diffusion and cell migration. These features can be tailored by the scaffold manufacturing process or by using covalent crosslinkers such as tannic acid [183], citric acid [184], and commercial products such as Kymene™ [185].

Scaffold biodegradation is essential for biomedical applications. Ideally, the material should be biodegraded at the same rate as the new tissue grows. Although most species *in vivo* do not have enzymes that hydrolyze nanocellulose, hence, exogenous enzymes can be administered to overcome such limitations. Nanocellulose-based materials have been extensively utilized as scaffolds for biomedical applications because they are biocompatible, show low cytotoxicity, and have the right wettability for cell functions [186,187]. Moreover, the high water content gives wet material a structural similarity to the extracellular matrix [188]. In the next section we review examples of CNC- and CNF-based structures in biomedical applications.

### 4.1 CNC in regenerative medicine

**Fig. 6:**
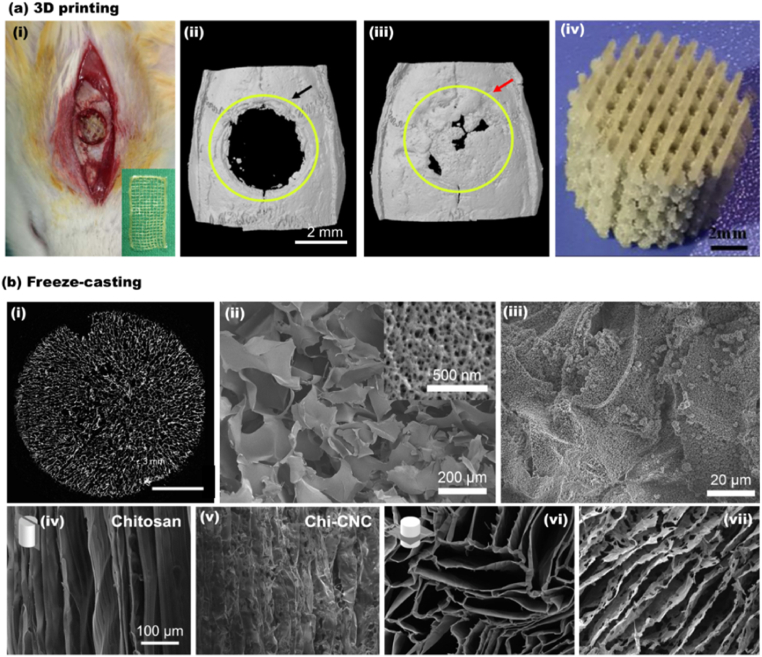
CNC containing 3D composites generated by 3D printing and freeze-casting. (a) 3D printed composite: (a_i_) The digital photograph of *in vivo* surgical examination with 3D printed CNC containing composite (CNC/Alg/Gel). The bone regeneration after 3 weeks in rat calvaria defect model. (a_ii_) the negative control, and (a_iii_) the healed section [189]. (a_iv_) High fidelity 3D printed PLLA/CC composite [191]. (b) The 3D structures obtained by freeze-casting. (b_i_) A 2D slice of macroporous CNC structure. (b_ii_) The cross-section SEM image of the macroporous aerogel at low and higher magnification (indicating the mesoporous CNC flakes). (b_iii_) HA formation and coverage of CNC aerogel after CaCl_2_ pre-treatment and submersion in SBF for 7 days [122]. SEM images of a longitudinal cross-section of (b_iv_) chitosan and (b_v_) chitosan-CNC scaffold frozen at the cooling rate of 10 °C/min. The SEM images of a transverse cross-section of (b_vi_) chitosan and (b_vii_) chitosan-CNC froze at a similar cooling rate of 10 °C/min (a_i-iii_) Adapted from Ref. [189] with permission of Copyright 2021 Elsevier. (a_iv_) Adapted from Ref. [191] with permission of Copyright 2020 Elsevier. (b_i-iii_) Adapted from Ref. [122] with permission of Copyright 2019 Elsevier. (b_iv-vii_) Adapted from Ref. [192] with permission of Copyright 2019 American Chemical Society.

CNC has been employed to reinforce polymer matrices and to fabricate functional nanocomposites for bone and cartilage tissue engineering, dental composites, soft tissues, drug carriers, and wound healing applications [88]. 3D printing is particularly useful for fabricating customized tissue constructs, namely, simple biomimetic devices such as tissue-like materials (*i.e.*, skin, bone, and cartilage). Also, the shear-induced alignment during hydrogel 3D printing facilitates the production of structures with controlled properties such as porosity and mechanical strength [98,102]. Such materials have been successfully fabricated and commercialized. Recently, Dutta et al. [189] developed 3D scaffolds based on alginate, gelatin, and CNC for bone tissue engineering. The CNC addition significantly increased the mechanical performance, mineralization efficiency, and the expression of osteo-specific gene markers. As shown in Fig. 6ai-iii, *in vivo* experiments with rat models demonstrated fast and high-density bone regeneration after three weeks of implantation. Patel et al. [190] prepared 3D printed compositions of alginate, gelatin, and CNC and achieved similar outcomes. Of relevance is CNC as a bio-based nucleation agent for bone repair purposes. For instance, Shuai et al. [191] demonstrated that the abundant hydroxyl groups in CNC interact with the poly-l-lactide (PLLA) molecular chains to increase the crystallization of the structure. Selective laser sintered of CNC/PLLA 3D scaffolds exhibited high fidelity (Fig. 6aiv), excellent biocompatibility, enhanced compressive modulus (351%), and strength (191%) compared with PLLA.

The freeze casting method has been used to prepare biomedical materials based on CNC. Scaffolds based on surface-modified CNC with sulfate (S) and phosphate (P) half ester group were developed by freeze-casting for bone tissue engineering. S–CNC freeze-casted scaffold was 89% porous, while P–CNC presented slightly less porosity (83%). Both CNC porous scaffolds were viable with Saos-2 cells and displayed hydroxyapatite growth in two weeks (Fig. 6bi-iii). The sulfated freeze-casted scaffolds demonstrated about 50% higher bone volume fraction than P–CNC after 12 weeks of implantation in Long Evans rats [122]. In another example, Yin et al. [192] studied equal mixed ratios of biocompatible chitosan and CNC for fabricating freeze-casted scaffolds for tissue engineering. The porosity, alignment of the pores, and mechanical performance were tunable by controlling the cooling rate during freeze-casting (Fig. 6biv-vii). It was also shown that blends of CNF and CNF improved the mechanical performance compared with that produced with neat CNC [192].

Other than 3D printing and freeze casting, CNC containing cast functional samples have been reported for biomedical applications. Athinarayanan et al. [193] developed HA/CNC nanocomposite from *Phoenix dactylifera* biomass and eggshell. HAP 10–50 nm crystals surrounded the 100–200 nm CNC and the structures exhibited nontoxic and biocompatible properties. This investigation highlights biomass valorization towards functional bioactive structures that can regenerate and rejuvenate bone microstructure. Al-Sabah et al. [194] studied the cast alginate/nanocellulose compositions for cartilage tissue engineering to study the impact of sterilization and CaCl_2_ crosslinking on the overall network construction and organization. The pore size enlarged, and the overall porosity decreased when the crosslinker concentration increased from 0.1 M to 1 M. Although the UV, autoclave, and ethanol immersion sterilization techniques demonstrated negligible differences in porosity and pore size, the Alginate/CNC composites displayed more than two times higher modulus when sterilized in ethanol.

**Fig. 7:**
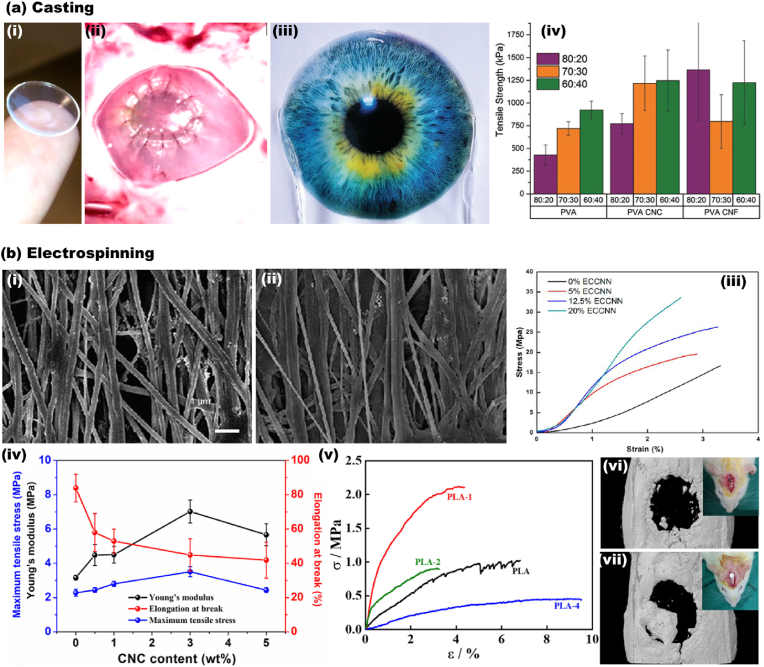
CNC containing 2D composites produced by casting and electrospinning. (a) Casted structures. (a_i_) Casted PVA/CNC transparent hydrogel sheet in the form of self-standing contact lenses. (a_ii_) Ex vivo implantation of the contact lenses to the porcine cornea [196]. (a_iii_) Coverage of the transparent CNC/CNF sheet on the lower half of the printed image. (a_iv_) The mechanical performance of the CNC/CNF sheet compositions and solvent ratios (DMSO/water) [195]. (b) Electrospun 2D composites. SEM images of electrospun cotton/CNC composites in (b_i_) 0% CNC and (b_ii_) 20% CNC. (b_iii_) The stress-strain curve demonstrating the mechanical performance of cotton/CNC electrospun structures [197]. (b_iv_) The mechanical performance of electrospun PBS/CNC bio-nanocomposite in various CNC concentrations of (0–5 wt%) [198]. (b_v_) The stress-strain curve of electrospun PLA/CNC composite containing varying CNC amounts (0–4 wt%). In vivo surgical examination after 3 weeks in rat calvarial defect for the (b_vi_) negative control and the (b_vii_) repaired sample [199]. (a_i-ii_) Adapted from Ref. [196] with permission of Copyright 2016 American Chemical Society. (a_iii-iv_) Adapted from Ref. [195] with permission of Copyright 2017 Royal Society of Chemistry. (b_i-iii_) Adapted from Ref. [197] with permission of Copyright 2014 American Chemical Society. (b_iv_) Adapted from Ref. [198] with permission of Copyright 2018 Elsevier. (b_v-vii_) Adapted from Ref. [199] with permission of Copyright 2020 Elsevier.

In other uses, Tummala et al. [195,196] fabricated high water content (93%) CNC-PVA contact lenses, Fig. 7ai-iv. The CNC reinforced contact lenses demonstrated excellent properties, including optical transparency (>95%), hyperelastic rubber-like mechanical performance, oxygen permeability (66 × 10^−11^ [(cm^2^/s) (mL O_2_/mL × mm Hg)]), and biocompatibility. The properties of the obtained soft tissue mimicked the cornea’s constituents and were comparable with commercial contact lenses.

Biomedical ultrathin fibers based on CNC have been developed through the electrospinning technique. Zhou et al. [155] prepared electrospun nanofibrous maleic anhydride grafted poly(lactic acid) (MPLA) reinforced with CNC. First, MPLA was prepared, and then freeze-dried CNC was added to a mixture of chloroform and DMF (volume ratio 8/2). The authors observed that the addition of CNC (5 wt%) increased the material’s thermal stability (heat resistance) and tensile strength while decreasing *in vitro* polymer degradation. Moreover, such nontoxic scaffold was able to support cell proliferation. He et al. [197] fabricated CNC reinforced all-cellulose nanocomposites by an electrospinning device equipped with a rotating drum to obtain aligned nanofibrils, Fig. 7bi-ii. The addition of 20% of CNC to the cellulose dope (cotton dissolved in LiCl/DMAc) improved the elastic modulus and tensile strength by 171% and 101%, respectively (Fig. 7biii). Human dental follicle cells exhibited high viability to the electrospun scaffold and proliferated on both microstructure’s surface and bulk.

Huang et al. studied the CNC reinforcement of polybutylene succinate (PBS) electrospun nanocomposites. The addition of 3 wt% CNC improved the hydrophilicity of the nanocomposite and enhanced the modulus, tensile strength, Fig. 7biv. The PBS/CNC scaffolds were viable with fibroblast cells, and nanocomposites biodegradation increased from 4.5% for pure PBS to more than 13% for the CNC-containing sample. Patel et al. [199] studied the potential of poly(lactic acid) and CNC compositions. The strong interaction of CNC and polymer chains resulted in higher thermal stability and mechanical properties (Fig. 7bv). Also, cell adhesion and proliferation significantly improved, and a higher expression of osteogenic gene markers was noticed for the composite. After 3 weeks, the *in vivo* result confirmed the potential of PLA/CNC as osteoconductive biomaterial for bone tissue regeneration (Fig. 7bvi-vii).

Cellulose acetate (CA) has been used in cellulose-based materials by electrospinning [151,152]; however, post-processing such as saponification with NaOH/ethanol and/or freeze-drying may be required. Joshi et al. [152] prepared multilayered cellulose scaffolds for bone tissue engineering. A 2D electrospun mat was prepared and modified into a 3D cellulose sponge using gas foaming, which allowed the biomimetic mineralization of the material and enhanced cell proliferation and attachment. In the method proposed by Li et al. [151], electrospun cellulose nanofibers (CelluNF) were first fabricated using CA, and then their surface was modified by a phosphorylation reaction. The authors observed the formation of HA covering the nanofibers, showing potential for bone tissue engineering. Table 2 summarizes some of the recent efforts in CNC-based structures developed for regenerative medicine produced by 3D printing, freeze-casting, casting, and spinning.

**Table 2:** Porous materials based on cellulose nanocrystals (CNC) and applied in regenerative medicine.

Materials	Fabrication Method	Cell type	Application	Experimental conditions	Observations	Ref.
CNCa)	3D printing	–	Tissue engineering	Nozzle diameter of 410 μm (22G), a flow rate of 70–120%, and a printing speed of 10–80 mm/s	Shear-induced alignment oriented the nanocrystal from 61 to 76% in the extrusion direction. Pore size: between 80 and 2125 μm.	[200]
CNC-chitosan	3D printing	MC3T3-E1 pre-osteoblast	Bone tissue engineering	Nozzle diameter 610 μm (20 G), Extrusion pressure 12–20 kPa. Cell density 5 million/mL. The bioprinted scaffolds were incubated in DMEM in a 24-well tissue culture plate at 37 °C for 24 h.	The degree of shrinkage ranged between 30 and 34%. The scaffolds enhanced the osteogenic differentiation and collagen formation in ECM.	[201]
PLLA-CNC	3D printing	MG-63 human osteosarcoma	Bone tissue engineering	The selective laser sintering optimized parameters were the spot size ∼200 μm, the laser power 7 W, the scanning speed 200 mm/s, and layer thickness of 150 μm.	The addition of 3 wt% CNC (nucleating agent) enhanced the compressive strength and modulus by 190% and 350%.	[191]
Alginate-gelatin-CNC	3D printing	human bone-marrow-derived mesenchymal stem cells	Bone tissue engineering	Nozzle diameter 200 μm (27 G), printing pressure of 80–600 kPa, printing speed of 8 mm/s, printing temperature of 30 °C, print-bed temperature 6 °C	Effective load transfer from the polymer matrix to CNC (due to the larger interconnected network) significantly improved the mechanical performance.	[190]
Alginate-gelatin-CNC	3D printing	Human bone marrow-derived mesenchymal stem	Tissue engineering	Cellulose nanocrystals (CNCs) from cotton pulps (10–20 nm width, 50–400 nm length, 12% solid content, crystalline index >70%), Nozzle diameter 200 μm (27 G), printing pressure of 100–500 kPa, printing speed of 5 mm/s, printing temperature of 35 °C, print-bed temperature 4 °C	CNC containing structures display enhanced mineralization efficiency and cell proliferation compared with the control sample	[189]
PEGDA-CNC	3D printing	–	Tissue engineering	The digital light processing parameters included the projector intensity of 18 mW/cm^2^, the exposure time of each layer was 4 s, the thickness of each cured layer (curing layer thickness) was set at 100 μm	The properties of digitally light processed 3D composites can be tailored by curing time and layer thickness	[202]
Gelatin-bioactive glass- CNC	Freeze casting	L929-fibroblast	Bone tissue engineering	The structure was put into a refrigerator. under −20 °C for 24 h, and then lyophilized at −57 °C and 0.05 bar for 48 h	The addition of CNC, even in small amounts, has a considerable effect on the mechanical performance	[203]
Cross-linked CNC	Freeze casting	Saos-2	Bone tissue engineering	All suspensions were frozen at −4 °C overnight to turn the suspension into a cryo-gel. The cryo-gels were then transferred into anhydrous ethanol for 5 days to form alco-gels. The alco-gels were placed inside of a critical point dryer and solvent exchanged with supercritical CO_2_ and gradually depressurized to ambient conditions to produce CNC aerogel.	No collapse of cryo-gels was observed during the solvent exchange, thereby making incremental ethanol exchanges unnecessary. Formation of hydroxyapatite layers, the proliferation of bone-like cells *in vitro*, and bone regeneration *in vivo*	[122]
Hydroxyapatite–CNC–silk fibroin	Freeze casting	MC3T3-E1	Bone tissue engineering	The scaffolds were freeze-dried for 24 h in 48-well plate. Then the scaffolds were immersed in 90% (v/v) aqueous methanol solution for 30 min to induce a structural transition that generated the water-insoluble scaffolds.	The average pore size and porosity of the scaffolds were 110 ± 7.3 mm and 90 ± 6.2%, respectively. The calvarial bone defect in rat was healed during 12 weeks of scaffold implantation	[204]
PVA-ovalbumin–CNC– HA	Freeze casting	–	Bone tissue engineering	The polymer solution was poured into 24-well plate, and freezed at −40 °C for 12 h, followed by drying under vacuum at similar temperature for 5 days.	Most optimal composition PVA/OVA/CNCs/n-HA (20:05:10:15) exhibited promises for short term bone regeneration	[205]
Alginate- gelatin- CNC	Freeze casting	Mesenchymal stem	Cartilage tissue engineering	The scaffolds were freeze-dried at −75 °C for 24 h	Nanocomposites with 96% porosity with a modulus of 0.5 GPa (higher than natural cartilage)	[206]
CNC/CNF-alginate	Freeze casting	L929-fibroblast	Tissue repair and wound healing	The samples were frozen in liquid nitrogen (−196 °C) for 5 min, followed by a freeze-drying step at −50 °C for 48 h. The dried materials were then added to a bath of CaCl_2_ at 2 wt % for 24 h. Then, the gels were washed with distilled water and were frozen with liquid nitrogen and freeze-dried again.	ECM biomimetic structure with promising mechanical properties, bioadhesion, cytocompatibility	[207]
CNC/CNF-chitosan	Freeze casting	–	Tissue regeneration	The slurry was poured in a polytetrafluoroethylene tube and was sealed with a copper mold (bottom section). The mold was then placed on liquid nitrogen. The molds were equilibrated to 4 °C for 10 min before a cooling rate of either 10 or 1 °C/min was applied until the mold reached a temperature of −150 °C. The frozen slurries for 72 h at 0.008 mbar and a coil temperature of −85 °C.	Obtaining structures with high porosity and surface area with controllable pore alignment	[192]
CNC/CNF-PVA	Casting	Human corneal epithelial	Soft contact lenses and cornea regeneration implants	The cast gels were placed at −20 ᵒC for 24 h followed by at least 48 h dialysis with deionized water.	Structures with higher water content and biocompatibility compared with commercial contact lenses	[195,196]
Dental glass ionomer cement- CNC	Casting	–	Dental composites		In addition to 0.4 wt% CNC, the compressive strength and tensile strength improved by 110% and 161%, respectively.	[208]
Crosslinked CNF/CNC-alginate	Casting	Human naso-septal chondrocytes	Cartilage tissue engineering	Crosslinking was performed at room temperature using 0.1 M, 0.5 M or 1.0 M calcium chloride (CaCl_2_).	Different crosslinking and sterilization conditions had a considerable impact on the microstructure architecture.	[194]
PCL-CNC	Casting and extrusion	Mouse preosteoblast	Bone tissue engineering	Prior to extrusion the caster films were placed in vacuum oven at 40 °C for 24 h	By the addition of 10 wt% CNC, the stiffness was doubled, and the ultimate tensile strength increased by 60%	[209]
HA-CNC	Casting	human mesenchymal	Bone tissue engineering	The hybrid structure was dried at ambient condition	The CNC-based nanohybrids from agro-waste were biocompatible, nontoxic, and it enhanced the calcium nodule growth	[193]
Cellulose acetatepropionate-CNC	Casting	–	Tissue engineering	After casting the suspension, a 0.3 T magnetic field was applied for 1 h at room temperature and the structures were kept for another 1 h without the magnetic field	Alignment of CNC under weak magnetic field effectively enhanced the mechanical performance (even at 0.2 wt% CNC addition)	[210]
Rosin-g-CNC	–	–	Antimicrobe structures	–	Strong and medium antibacterial activity was observed against Gram-negative and Gram-positive bacteria, respectively	[211]
Porphyrin-CNC	–	M. smegmatis, E. coli and S. aureus bacteria strains	Antimicrobe structures	–	Development of photobacterial materials with high efficiency against Gram-negative, Gram-positive, and mycobacterium	[212]
Polyrhodanine-CNC	–	HeLa (ATCC CCL-2)	Antimicrobe structures	–	Core-sheath antimicrobial nanoparticles with killing efficiency of over 95% *E.coli* and *B. subtilis*	[213]
CNF–CNC	Electrospinning	Human dental follicle	Artificial organ	The operating voltage of 20 kV, flow rate 0.03 mL/min, A steel rotating collector (6 cm in diameter) wrapped with aluminum foil was placed 10 cm away. The tangential velocity of the collector was set at 300 m/min	All cellulose nanocomposite with high fiber alignment.	[197]
PBS-CNC	Electrospinning	3T3 fibroblast	Tissue engineering	The operating voltage of 20 kV, flow rate 1.0–2.0 mL/h and the distance between the electrodes was 18 cm.	The *in vitro* degradation displayed to increase from 4.5% for pure PBS to about 14% for PBS-CNC (3 wt% CNC) during 28 days.	[198]
PLA	Electrospinning	–	Short-term applications in the tissue engineering	The operating voltage of 15 kV, flow rate 1.5 mL/h and the distance between the electrodes was 20 cm. The obtained fibers were collected as mats, and were vacuum-dried at 80 °C for 24 h.	Improvement in heat resistance, tensile stress, young’s modulus, In vitro degradation	[153]
PAN-CNC	Electrospinning	–	Dental composites	The operating voltage of 17.2 kV, flow rate 2.0 mL/h and the distance between the electrodes was 20 cm.	The addition of 3 wt% CNC resulted in a significant increase in flexural and fracture strength.	[214]
PLA-CNC	Electrospinning	Human bone marrow-derived mesenchymal stem	Bone tissue engineering	The operating voltage 16 kV, distance between the electrodes 15 cm, and rolling speed of the collector was 2000 rpm.	Excellent biocompatibility and promising osteoinductivity was obtained according to *in vivo* studies during three weeks	[199]
PEG-g–CNC–PLA	Electrospinning	Human mesenchymal stem cells	Bone tissue engineering	The operating voltage of 20 kV, flow rate 0.5 mL/h and the distance between the electrodes was 15 cm, and 18 G blunt stainless-steel needle. The obtained fibers were collected as mats and were vacuum-dried at 80 °C for 24 h.	The addition of PEG improved the biocompatibility of the composite	[215]
MAH-g-PLA	Electrospinning	Adipose-derived mesenchymal stem	Bone tissue engineering	The operating voltage of 15 kV, flow rate 1.5 mL/h and the distance between the electrodes was 20 cm, The obtained fibers were collected as mats and were vacuum-dried at 80 °C for 24 h.	Improvement in heat resistance and tensile strength. Reduction *in vitro* degradation rate. Capable of supporting cell proliferation.	[155]
PCL-CNC	Electrospinning	–	Controlled drug delivery	The operating voltage of 17 kV, flow rate 0.9 mL/h and the distance between the electrodes was 16 cm.	Addition of CNC enhanced the tensile strength and modulus by 46% and 47%	[216]
Alginate- gelatin- CNC	Injectable hydrogel	3T3 fibroblast/MC3T3-E1 osteoblast	Bone regeneration	Injection with 18 G nozzle followed by the addition of 10.5 mL 0.05 M ZnSO_4_ (ionic crosslinking).	The presence of CNC enhanced the hydrogel/cell interactions	[217]

a) CNC, cellulose nanocrystal; PLLA, Poly-l-lactic acid; PEGDA, Poly(ethylene glycol)diacrylate; PVA, Polyvinyl alcohol; HA, Hydroxyapatite; CNF, Cellulose nanofibrils; PCL, Polycaprolactone; PBS, Polybutylene succinate; PLA, Polylactic acid; PAN, Polyacrylonitrile; PEG, Polyethylene glycol; MAH, Maleic Anhydride. ECM, extracellular matrix.

### 4.2 CNF in regenerative medicine

**Fig. 8:**
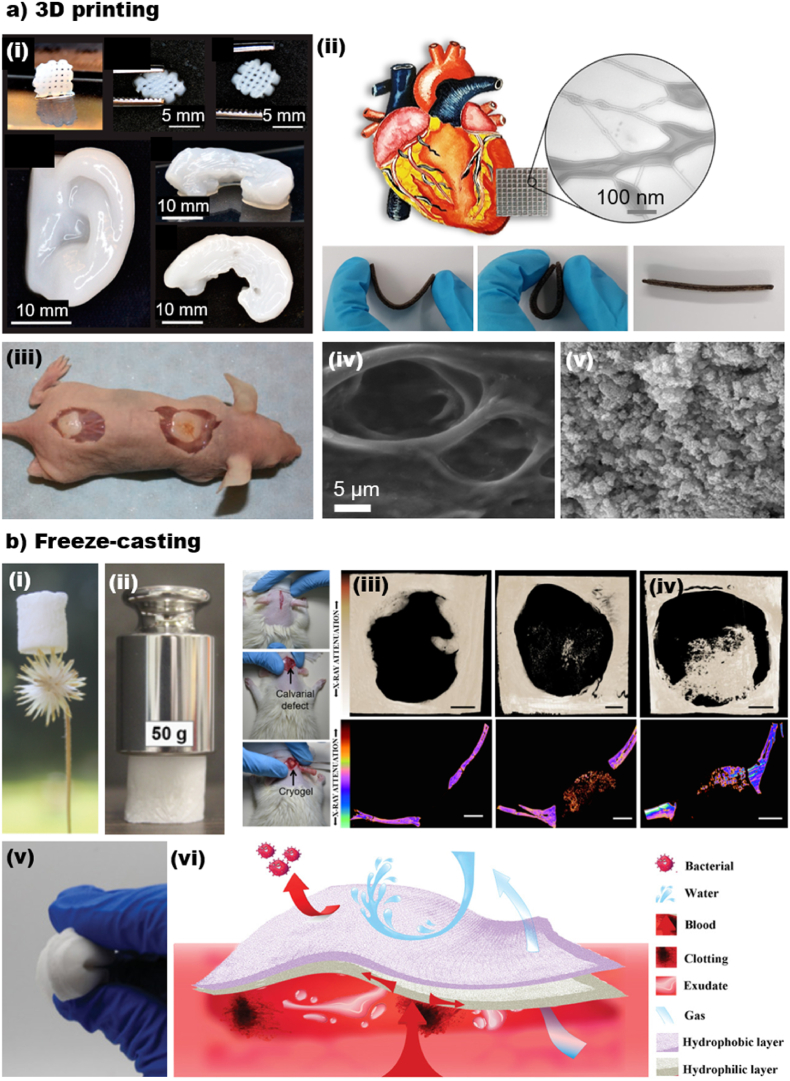
CNF containing 3D composites generated by 3D printing and freeze-casting. (a_i_) 3D printed small grids (7.2 × 7.2 mm^2^) with shape recovery behavior, 3D printed human ear, and 3D printed sheep meniscus [112]. (a_ii_) Electrically conductive, elastic, drug-loaded cardiac patch based on TOCNF/PGS/PPy. Shape recovery and flexibility of the cardiac patch [232]. (a_iii-iv_) The SEM images of CNF-based structure (a_iii_) before and (a_iv_) after mineralization for bone tissue engineering application [233]. (b_i-ii_) Light-weight and strong composite based on CNF and bioactive glass (CNF/BG) for bone tissue engineering. The (b_iii_) negative control and the (b_iv_) the rat calvarial regeneration after 56 days of scaffold implantation of CNF/BG [225]. (b_v-vi_) Janus flexible sponges based on CNF for wound healing application and treatment of hemorrhagic wounds [234]. (a_i_) Adapted from Ref. [112] with permission of Copyright 2015 American Chemical Society. (a_ii_) Adapted from Ref. [232] with permission of Copyright 2020 Wiley-VCH. (a_iii-iv_) Adapted from Ref. [233] with permission of Copyright 2018 American Chemical Society (b_i-iv_) Adapted from Ref. [225] with permission of Copyright 2019 Royal Society of Chemistry. (b_v-vi_) Adapted from Ref. [234] with permission of Copyright 2020 Wiley-VCH.

CNF-based structures have been reported for regenerative medicine. CNF aqueous suspension is similar to the extracellular matrix (ECM) and demonstrates sufficient mechanical stability for secondary cell culture purposes, even in their pure form and with no addition of polymers/particles [[218], [219], [220]]. However, to obtain properties beyond biocompatibility and cell interaction, polymers or (nano)particles are added to CNF suspension to fabricate hybrid or composite materials, allowing the incorporation of additional functionalities or the enhancement of specific properties [221,222]. In this sense, nanocellulose can be combined with bioactive materials, such as metallic or magnetic (nano)particles [223], quantum dots [224], bioactive glass [225] and so on [226]. Such particles can be easily integrated into the nanocellulose matrix by surface functionalization, electrostatic interactions or simply mixing during the scaffold preparation process, providing unique functionalities, such as antimicrobial activity [227], imaging capabilities [228], and stimuli-responsive behavior [229,230]. Thus, the successful hybridization of nanocellulose with bioactive nanoparticles holds great promise for a wide range of biomedical applications. In this direction, Markstedt et al. [112] prepared 3D bioprinted shapes resembling cartilage tissues using a bio-ink consisting of CNF and alginate at room temperature and low pressure (critical features to work with living cells). The authors successfully printed an ear and a meniscus (Fig. 8ai) with good shape fidelity and showed biocompatibility and suitability for cell culture (human nasoseptal chondrocytes - hNC).

Ajdary et al. [114] combined poly(glycerol sebacate) (PGS), polypyrrole (PPy), and 2,2,6,6-tetramethylpiperidine-1-oxyl radical-oxidized nanocellulose (TOCNF) to prepare cardiac patches with good elasticity, flexibility, and electrical conductivity for treatment of cardiovascular diseases (Fig. 8aii). The samples were prepared by 3D printing, followed by freeze-drying. The scaffolds performed as a mechanical support for cells and promoted reorganization into functional tissue. Moreover, the slow degradation of the printed scaffold could prevent the burst release of drugs, which is suitable for long-term delivery for therapies. Kuzmenko et al. [231] fabricated other CNF-carbon nanotubes (CNT) conductive hydrogels for neural guideline application. In their work, shear-thinning inks of CNF/CNT were mixed and 3D printed to form thin guidelines of less than 1-mm diameter. The neural guidelines displayed electrical conductivity and exhibited high viability with neural cells.

Several studies have demonstrated the high potential of 3D printable CNF/alginate compositions for cartilage tissue engineering applications [112,194,207,233,[235], [236], [237]]. For instance, Möller et al. [236] bioprinted cell-laden CNF/alginate scaffolds to investigate the clinical relevance for chondrogenesis. The cell-containing samples retained their integrity up to 60 days *in vivo* and displayed potential for cartilage reconstruction application. However, cell-laden inks are usually affected by the imposed shear during the printing process, and the challenges to reproduce a structure with comparable mechanical properties to natural human cartilage still exist. Abouzeid et al. [233] studied crosslinked CNF/alginate compositions for bone tissue engineering. The equal mass ratio of CNF and alginate demonstrated compressive strength of 455 MPa (87 MPa for pure CNF) and modulus of 1511 MPa (135 MPa for pure CNF) after crosslinking. After 14 days of mineralization, the hydroxyapatite content of the scaffold was evaluated to be around 20% with the average crystal size of 25.4 nm (Fig. 8aiii-iv).

CNF freeze-casting has also been a facile approach to develop scaffolds for tissue engineering applications. For example, Carlström et al. [238] investigated compositions of crosslinked CNF and gelatin for bone tissue regeneration and tuned the biodegradation rate of gelatin when combined with CNF. The compositions exhibited higher mechanical properties and lower degradation rates. After crosslinking, the porosity decreased from ca. 90% for pure CNF to 70% for CNF/gelatin, while the surface area of the composite more than doubled.

Ferreira et al. [225] developed bioactive, biocompatible, and strong freeze-casted cryogels from CNF and bioactive glass for bone tissue regeneration (Fig. 8bi). The low-density cryogels comprised 80% bioactive glass and 20% CNF on a dry basis and resisted high compressive loads, up to over 1250 times cryogels' original weight (Fig. 8bii). The cryogels displayed remarkable performance *in vivo* with no damage to the excretory environment and effective bone formation during 56 days of the examination (Fig. 8biii-iv). Tang et al. [239] studied the compositions of CNF/polyethylene glycol diacrylate (PEGDA) for cartilage tissue engineering. CNF was performed as supporting material in their work and facilitated the structure with mechanical integrity and high porosity of up to 90% (porosity less than 10% in pure PEGDA). This enhancement in the porosity significantly impacted the cell viability and proliferation of the NIH 3T3 cells on CNF/PEGDA scaffolds.

Beyond bone and cartilage tissue engineering, CNF-based freeze-casted scaffolds have been applied for skin tissue regeneration and wound healing purposes. To illustrate, Ghafari et al. [240] produced CNF/polyvinyl alcohol (PVA) bilayers inspired from the dermis and epidermis in the skin top layers. The first skin mimic layer, dermis, comprised 0.4 wt% CNF and 0.1 wt% PVA, and the second layer, epidermis, was made from 1.4 wt% CNF and 0.35 wt% PVA. As in the actual skin, the first layer had less bulking density and higher water uptake. The overall porosity of dermis and epidermis biomimetic structures were more than 95% and 88%, respectively, and both layers provided a suitable microenvironment for fibroblasts and keratinocyte cells to adhere and proliferate. Cheng et al. [234] combined cross-linked CNF/organosilanes and chitosan to prepare Janus sponges, which showed high effectiveness for treating hemorrhagic wounds. The Janus sponges displayed a shape-memory and remarkable flexibility in wet conditions. Compared to the common gauze, the Janus sponges displayed 49% blood loss reduction, indicating potential for wound dressing (Fig. 8bv-vi).

As indicated earlier, electrospinning is a promising technique to obtain porous interconnected structures. CNF has been used as a bio-based reinforcing agent in combination with polymers such as PLA, PBS, etc. For example, Abudula et al. [241] produced PLA/PBS/CNF electrospun nanofibers for vascular tissue engineering purposes. The CNF addition tuned the nanofiber diameter to around 270 nm and resulted in more homogeneous fiber morphology than pure PLA and PBS. CNF-containing composites absorbed antibacterial enzyme lysozyme and exhibited a higher biodegradation rate in the presence of Proteinase K enzyme. Joshi et al. [152] investigated the saponified electrospun cellulose acetate nanofibers post-treated with sodium borohydride solution. The post-treatment enhanced the porosity and decreased the packing of the nanofibers to form 3D spongy scaffolds with enhanced hydroxyapatite mineralization and cell interactions. Table 3 summarizes the recent efforts in CNF/CNF-based structures developed for regenerative medicine.

**Table 3:** Porous materials based on cellulose nanofibrils (CNF) and applied in regenerative medicine.

Material	Fabrication Method	Cell type	Application	Experimental conditions	Observations	Ref.
CNF^a^ TOCNF Acetylated CNF	3D printing	H9c2 cardiomyoblast	Cell culturing scaffolds	Nozzle diameter of 410 μm, 630 μm, and 840 μm, extrusion pressure of 35–55 kPa, print speed 5–12 mm/s	High biocompatibility with cardio myoblasts and induced cell proliferation for 21 days	[218]
CNF-alginate- lignin particles	3D printing	HepG2 Hepatocellular carcinoma cell line	Soft tissue engineering	Nozzle diameter of 410 μm (22 G), print speed 11.5 mm/min. The scaffolds were crosslinked in 90 mM CaCl_2_, stored at ambient conditions for 2 h, or in 1 × DPBS + solution for 1 week at 4 °C	The shear-thinning behavior of CNF did not alter due to addition of up to 25% LPs. Good cell viability regardless of the lignin content (LP/CNF ratio 0–25 w/w %)	[242]
PGS-PPy-TOCNF	3D printing	H9c2 cardiomyoblast	Treatment of cardiovascular diseases	Nozzle diameter of 840 μm (18G). After 3D printing, the samples were frozen overnight at −18 °C followed by freeze-drying for 48 h, at −49 °C and 0.05 mbar. Then, the sample was cured for 48 h in a vacuum oven at 120 °C	Porosity of 78 ± 2%, electrical conductivity of 34 ± 2.7 mS cm^−1^, Young’s Modulus 0.6 ± 0.16 MPa, high biocompatibility with cardiomyoblasts and induced cell proliferation for 28 days	[114]
CNF-CNT	3D printing	HPACC, human neuroblastoma	Neural guidelines	Nozzle diameter of 300 μm, printing pressure 65 kPa, print speed 10 mm/s	Viability, proliferation, and attachment of cells to the guidelines with less than 1 mm diameter and 3.8 × 10^−1^ S cm^−1^ conductivity	[231]
Cross-linked TOCNF-alginate	3D printing	–	Bone tissue engineering	Nozzle diameter of 500 μm, printing pressure of 50 kPa, printing speed 16 mm/s. The structures were post-crosslinked in 0.5 M CaCl_2_ for 20 min.	Successful ex vivo mineralization of HA up to 20% in the scaffold made from an equal ratio of CNF and alginate. The compressive strength and elastic modulus ranged from 87 to 455 MPa, and 135–1511 MPa, respectively.	[233]
CNF	3D printing	–	Tissue engineering	Nozzle diameter 200–510 μm	4D shape-morphing mesoscale structures that are initiated upon hydration. Young’s Modulus in longitudinal and Transverse direction 1267 ± 201 and 1011 ± 39, respectively.	[121]
polyurethane-CNF	3D printing	NIH 3T3 mouse skin fibroblasts and human fibroblasts	Tissue engineering	Nozzle diameter 160 μm, Printing pressure 50–200 kPa, printing speed 7–10 mm/s	Waterborne PU and CNF developed high fidelity structures with high cell proliferation. The compression storage modulus decreased from 1.57 MPa at day 0–0.91 MPa at day 28.	[243]
Cross-linked CNF	3D printing	Mouse embryonic fibroblast	Cell culture scaffold		The smallest tested concentration of CaCl_2_ (0.22 wt%) was better for the cells. The higher stability of the structures by cooling and crosslinking steps that favor cell viability.	[220]
Cross-linked CNF-alginate	3D printing	Human nasoseptal chondrocytes	Cartilage tissue engineering	Nozzle diameter 300 μm, Printing pressure 20–60 kPa, printing speed 10–20 mm/s	Shapes resembling an ear and a meniscus with high fidelity and stability were successfully printed. Viability of 73% and 86% after 1 and 7 days of 3D culture.	[112]
CNF-alginate	3D printing	human nasal chondrocytes, human bone marrow-derived mesenchymal stem	Cartilage tissue engineering	Cell density 10 million/mL	Effective cartilage synthesis occurred in cell-laden 3D constructs with promising mechanical properties hand high fidelity.	[236]
CNF-alginate	3D printing	human nasal chondrocytes	Cartilage tissue engineering	Cell density 20 million/mL, nozzle diameter 150 μm	Excellent stability of shape and size that supports the redifferentiation of cells.	[235]
TOCNF- Aloe vera	3D printing	–	Tissue engineering	Nozzle diameter 630 μm	Development of fully bio-based hydrogels with high stability and excellent viscoelastic properties. Porosity higher than 80–95% and a high-water uptake capacity of up to 46 g/g. Tensile modulus of 4.95–73.44 kPa	[244]
CNF–CaCO3	3D printing	–	Controlled drug release	Nozzle diameter 1.21 mm	Achieving controlled drug release that mimicked the colons condition	[245]
CNF-alginate	3D printing	L929 mouse fibroblast	Wound healing	Nozzle diameter 580 μm, printing speed 3 mm/s	CNF from sugarcane bagasse residue has efficient biocompatibility	[237]
Cross-linked CNF-GelMA	3D printing	3T3 fibroblast	Wound healing	Precision tips (25G, and 30G), printing pressure 65–80 kPa, printing speed 16–32 mm/s	A facile approach to obtain high cell compatibility and proliferation. Mechanical strength in the range of 2.5–5 kPa	[246]
Cross-linked TOCNF	3D printing	Human dermal fibroblasts	Wound healing	Nozzle diameter 200 μm, printing pressure 50 kPa, printing speed 8 mm/s	Higher rigidity of the scaffold improves cell proliferation. Mechanical strength in the range of 3–8 kPa	[219]
CNF- Peptide	3D printing	–	Wound healing	Nozzle diameter 410–840 μm, printing pressure 100–350 kPa, printing speed 10 mm/s	Obtaining structures with programmable actuation and texture with controlled mechanical and antimicrobial properties	[247]
CNF- bioactive glass	Freeze casting	MC3T3-E1 cells	Bone tissue engineering		Light-weight bio-active cryogels that promote ion release (Si, Ca, P, Na). Cryogel compression strength range 11 ± 1 to 24 ± 1 kPa	[225]
Cross-linked gelatin-CNF	Freeze casting	Human bone marrow mesenchymal stem	Bone tissue engineering	The samples were frozen at −20 °C followed by freeze-drying for 24 h	Different crosslinking methods did not have an adverse biological effect on cells, and the composite promoted cell differentiation	[238]
CNF-PEGDA	Freeze casting	NIH 3T3 mouse embryonic fibroblast	Cartilage tissue engineering	The samples were frozen at −80 °C for 24 h followed by freeze-drying at −68 °C for 48 h	Structure with about 90% porosity and 1–3 MPa mechanical strength.	[239]
CNF-PVA	Freeze casting	Fibroblast cells and keratinocytes	Skin tissue engineering		A novel, integrated skin mimics bilayer structures (mimicking Epidermis and Dermis). Elongation at break range of 52 ± 7 to 91 ± 1. Young’s modulus range of 0.04–8.3 ± 1.8 kPa and porosity of 77 ± 7.3%	[240]
TOCNF	Freeze casting	U937 cell	Tissue engineering	The structures were frozen at −20 °C for 24 h and then freeze-dried for 24 h	Production of considerably less inflammatory cytokines than gelatin according to *in vivo* test	[249]
Cross-linked CNF/organosilanes and chitosan	Freeze casting	Human skin fibroblasts	Hemostatic dressing	The hydrophilic layer was frozen at −80 °C, and after permeation of the hydrophobic layer, the Combination structure was freeze-dried for 36 h at −50 °C.	Effective bleeding control with nearly 50% less blood loss	[234]
CNC/CNF-alginate	Freeze casting	L929-fibroblast	Tissue repair and wound healing	The samples were frozen in liquid nitrogen (−196 °C) for 5 min, followed by a freeze-drying step at −50 °C for 48 h. The dried materials were then added to a bath of CaCl2 at 2 wt % for 24 h. Then, the gels were washed with distilled water and were frozen with liquid nitrogen and freeze-dried again.	ECM biomimetic structure with promising mechanical properties, bioadhesion, cytocompatibility	[207]
CNC/CNF-chitosan	Freeze casting	–	Tissue regeneration	The slurry was poured in a polytetrafluoroethylene tube and was sealed with a copper mold (bottom section). The mold was then placed on liquid nitrogen. The molds were equilibrated to 4 °C for 10 min before a cooling rate of either 10 or 1 °C/min was applied until the mold reached a temperature of −150 °C. The frozen slurries for 72 h at 0.008 mbar and a coil temperature of −85 °C.	Obtaining structures with high porosity and surface area with controllable pore alignment	[192]
Crosslinked CNF/CNC-alginate	Casting	Human naso-septal chondrocytes	Cartilage tissue engineering	Crosslinking was performed at room temperature using 0.1 M, 0.5 M or 1.0 M calcium chloride (CaCl_2_).	Different crosslinking and sterilization conditions had a considerable impact on the microstructure architecture.	[194]
PLA-PBS- CNF	Electrospinning	Dermal fibroblasts	Vascular tissue engineering	The operating voltage of 20 kV, flow rate 0.5 mL/h and the distance between the electrodes was 12 cm.	ECM mimic microstructure with excellent cell proliferation and attachment on the composites	[241]
CNF–CNC	Electro-spinning	Human dental follicle	Artificial organ	The operating voltage of 20 kV, flow rate 0.03 mL/min, A steel rotating collector (6 cm in diameter) wrapped with aluminum foil was placed 10 cm away. The tangential velocity of the collector was set at 300 m/min	All cellulose nanocomposite with high fiber alignment and indentation modulus of 2492 ± 61.6 MPa	[197]
Cellulose acetate	Electro-spinning**	Osteoblast	Bone tissue engineering	The operating voltage of 17 kV, flow rate 1.0 mL/h and the distance between the electrodes was 15 cm. Then, the fabricated mats were dried at 40 °C for 24 h.	Biomimetic mineralization, enhanced cell proliferation, and attachment. The apparent density of 0.26 g/mL	[152]
Cellulose acetate	Electro-spinning	–	Bone tissue engineering		Formation of HA covering the nanofibers. Specific surface areas of the composite were 51.08 m^2^/g. The CelluNF/HAp composites had mesopores in a range of 2–18 nm, and large amount of micropores in a range of 1.03–2.0 nm	[151]

a CNF: Cellulose nanofibrils; TOCNF: TEMPO-oxidized cellulose nanofibrils; PGS: Poly (glycerol sebacate); PPy: Polypyrrole; CNT: Carbon nanotube; GelMA: Gelatin meth acryloyl; PEGDA: Poly(ethylene glycol)diacrylate; PVA: Polyvinyl alcohol; PLA: Polylactic acid; PBS: Polybutylene succinate; ECM: extracellular matrix.

In sum, nanocellulose-based scaffolds have been fabricated by relatively simple processes and were successfully used to engineer biomimetic tissue-like constructs for regenerative medicine. In general, the scaffolds are prepared to mimic the structural morphology of the native tissue of interest, *i.e.*, both tissue architecture and biological composition. A critically important process for this purpose is the increase in the bioactivity of the scaffolds to enhance their interaction with the surrounding tissue. This can be achieved from the combination of nanocellulose and bioactive materials [225], as the efficacy of this combination has governed the nanocellulose-based scaffold. Another critical issue to consider is the different stages that engineered materials often need to go through, mainly associated with cell distribution within the scaffolds, biodegradation (*i.e.*, spatial- and temporal-controlled biodegradation), and engineered tissue integration. When live cells (or bioactive materials) are used, their viability must be considered at the end of the process.

### 4.3 Translation from academic research and commercial viability

The translation of nanocellulose-based biomedical scaffolds into products and use in patients are challenging and should involve standardized developmental frameworks comprising responsible and reproducible practices. Efforts in this area are subjected to strict regulatory processes that vary according to the different countries/regions jurisdictions intended to be marketed [250]. Awareness of the products' developmental frameworks and regulatory requirements, since the early stages of their development, is essential to guarantee compliance with efficacy and safety standards necessary for future marketing authorization [250]. This section brings a guideline for the responsible development of tissue engineering scaffolds and the current regulatory frameworks in the United States. Briefly, the tissue engineering scaffold needs to undergo three stages before launching onto the market: (a) product discovery, (b) clinical research, and (c) preapproval request [250].

***Product discovery***. The motivation to create a new scaffold should always be to address unsolved clinical needs. A coordinated process of co-creation involving different stakeholders (patients, clinicians, companies, policy makers, and other researchers) is necessary to ensure a complete understanding of the current clinical practice to identify clinical situations that require improvement [251]. Once a suitable unmet clinical need is identified, the researchers should raise a series of research questions to guide their chosen materials to solve the identified need. In this regard, nanocellulose-based scaffolds have been proposed for a wide range of applications covering bone grafting, antimicrobial treatment, cartilage regeneration, dental applications, drug delivery systems, wound healing, skin repair, and constitute an attractive alternative.

Following the choice of which tissue engineering scaffold should be developed, the next step requires an iterative process for product refinement that should involve rounds of material characterizations to guarantee that the generated product presents the desired bulk and surface characteristics for safe and effective implantation. Implant safety is dependent on the property of biocompatibility, which represents the primary requirement for further marketing authorization [252]. The pre-clinical biological safety evaluation of scaffolds should be performed in a cyclical manner encompassing the material manufacture and four stages: (Stage 1) material chemical characterization, (Stage 2) toxicological risk assessment, (Stage 3) *in vitro* testing, and (Stage 4) *in vivo* testing. If results are uncertain or the material fails to demonstrate biocompatibility, the process should re-start with a reformulated material. The biological safety evaluation of tissue engineering scaffolds should be standardized and follow responsible and reproducible methodologies. The most reliable way to meet high standards during this process is to follow standardization bodies such as the International Organization for Standardization (ISO) guidelines. ISO Standards are internationally agreed standards produced by experts that describe the optimal way to perform a wide range of activities related to quality management, environmental management, health and safety standards, *etc*. [253]. In this context, the ISO 10993 constitutes a set of standards for evaluating the biocompatibility of medical devices.

*Stage 1 – Standardized chemical and structural characterization of nanocellulose scaffolds*. The characterization of scaffolds should consider the leachable/extractable products released by its dissolution, its internal structure, and surface characteristics. The complexity of this characterization may vary depending on the nature of the scaffold. The common standards used to support effective and reproducible characterization of biomaterials are listed next. ISO 10993–12:2021 [254] gives guidance on sample preparation and the selection of reference materials for medical device testing. ISO 10993–18:2020 [255] provides a framework to recognize biomaterial constituents, allowing identifying possible biological hazards from material constituents. ISO/TS 10993–19:2020 [256] brings a set of test methods that can be used to identify and evaluate the physic-chemical, morphological, and topographical properties of biomaterials.

*Stage 2 – Standardized toxicological risk assessment*. Once the scaffold’s structure and chemical composition are well understood, the next step in evaluating its biological safety is to perform a detailed toxicological risk assessment. This assessment should consider the extent of a patient’s potential exposure to hazardous chemicals previously identified in stage 1 and, when possible, determine a dose-response relationship. The level of risk is determined by combining this information and should be based on worst-case exposure scenarios. Standardized procedures for toxicological risk assessments can be found in ISO 10993–17:2002 [257]. This standard specifies a systematic process through which risks are rising from toxicologically hazardous substances in biomaterials can be quantified. This ISO was last reviewed in 2017 and will be replaced by ISO/CD 10993-17.2, ‘Biological evaluation of medical devices — Part 17: Toxicological risk assessment of medical device constituents' (under development) [258]. If a scaffold passes this evaluation, it requires no further tests, and the biological safety evaluation should be considered complete. On the other hand, *in vitro* tests should be performed in case the results are uncertain.

*Stage 3 – Standardized in vitro testing*. The *in vitro* biocompatibility of a scaffold is most easily accessed through testing the effect of the chemical leachable products that may be released from the material, but other approaches may be relevant depending on the type of the scaffold. Nanocellulose scaffolds usually present physical characteristics and 3D architecture that resemble the fibrous phase of the ECM. Thus, the scaffold capacity assessment to support cell attachment and proliferation is essential. It is expected that their dissolution products will be released and affect cells locally and may transit systemically, affecting positively or negatively other cell types. Thus, the *in vitro* investigation of the scaffolds' cytocompatibility to the relevant cell types is essential.

*In vitro* models represent simplified versions of specific natural phenomena and should be used to isolate and investigate particular potential clinical issues such as cell adhesion, cytocompatibility, hemolysis (ISO 10993–4:2017 [259]), genotoxicity, carcinogenicity, and reproductive toxicity (ISO 10993–3:2014 [260]). They should never be considered as a replica of the full complexity of any natural system [261]. Within their limitations, engineered *in vitro* models draw inspiration from human tissues to respond to scaffolds. A detailed description of the different types of *in vitro* models and their roles in improving research quality in tissue engineering and reducing animal use can be found elsewhere [261].

In responsible practice, ISO/IEC 17025 is the international reference for testing and calibration laboratories. Such ISO enables laboratories to demonstrate that they operate competently and can generate valid results. Using laboratories that possess ISO/IEC 17025 [262] accreditation increases the credibility of the biological evaluation of materials and facilitates collaboration with other research centers and acceptance by regulatory agencies. Using accredited laboratories to investigate the safety and effectiveness of proposed novel biomedical scaffolds complies with responsible and reproducible practice and facilitates the acceptance by regulatory bodies for marketing authorization.

*Stage 4 – Standardized in vivo testing*. The *in vivo* assessment of tissue compatibility of a scaffold should aim to investigate if the material performs as intended and presents no sign of significant local or systemic toxicity to the recipient. The scaffolds and their components can be categorized by the type of contact with living tissue (superficial contact, partly implanted, totally implanted) and the duration of the contact (*e.g.*, seconds, minutes, hours, days, months, years) to select the appropriate tests. According to ISO 10933, the biological safety of all biomedical materials should include tests of cytotoxicity, sensitization, and irritation. Further *in vivo* tests can vary according to the end-user application and to the type of biomaterial. In this regard, nanocellulose-based scaffolds used for regenerative medicine should constantly be tested for their local and systemic toxicity.

Responsible practice in using animals for research implies following strict ethical procedures established by well-recognized international organizations such as the Association for Assessment and Accreditation of Laboratory Animal Care (AAALAC) [263]. This association promotes the humane treatment of animals in science through voluntary accreditation and assessment programs and seeks to accommodate scientific progress within the scope of animal well-being. Using laboratories accredited by AAALAC to investigate *in vivo* efficacy and safety of scaffolds for regenerative medicine helps guarantee high standards and reproducibility of results while complying with ethical principles of animal care, which will significantly strengthen the further application for marketing authorization.

***Clinical Trials for tissue engineering scaffolds***. Clinical trials might be necessary depending on the results obtained in the previous stages of development and how the regulation body classifies the product that is intended to be commercialized. A detailed description of the three regulatory classes of medical devices established by the Food and Drug Administration (FDA) is presented in the next section. Awareness about the correct classification of the proposed novel scaffolds is essential for the decision-making process for preapproval requests.

Clinical trials are studies involving human participants. They are intended to investigate if a particular treatment is safe and how well it works compared to other pre-existing treatments. The particular design of a clinical trial is subject to the results observed in the previous experimental stages and the available literature about similar materials and usually is consisted of one to four phases. Phase I trial is conducted in a reduced number of participants who can either be healthy (who are given payment compensation) or ill individuals. The reduced number of participants enables all of them to be closely monitored for any side effects. This phase aims to get an idea of how safe the treatment is. In phase II trials, the treatment is tested in a more significant number of participants to investigate possible side effects better, and another treatment may or not be used as a comparison. Phase III trials usually involve hundreds or thousands of patients. Its main goal is to compare the efficacy of the new treatment with existing treatments. In this phase, the study should be randomized and double-blinded, *i.e.*, the patients' allocation in each treatment group should be randomized, and neither the patient nor the investigator should know to which group each participant belongs. This study design has been observed to be the most reliable and accurate to compare treatments in clinical trials.

After phase III trials, all the data collected on the scaffold’s safety, quality, and efficacy need to be organized in a dossier called Common Technical Document (CTD). This document is the standard format of application for marketing authorization (MA). A specific regulatory agency will evaluate it depending on the jurisdiction in which the proposed regenerative scaffold is to be commercialized once every nation has different regulatory frameworks and associated specific legislations with evaluating tissue engineering scaffolds. Phase IV trials are only carried out after the new treatment is effective and given a license. In this phase, investigators should keep track of post-marketing reported adverse effects, how well the treatment works in a much larger number of people, the long-term risks and benefits, and any possible rare adverse effects. An exciting way to design effective clinical trials to test new nanocellulose-based scaffolds for tissue engineering is to look for previous clinical trials on similar materials on the website ClinicalTrials.gov [264]. This platform is provided by the U.S. National Library of Medicine and contains information about more than 377,847 clinical studies carried out in more than 220 countries. The following section describes the regulatory framework for tissue engineering scaffolds to be marketed in the United States of America.

***Pre-market regulations for tissue engineering scaffolds***. In order to be marketed, a tissue engineering scaffold, including nanocellulose-based scaffolds, needs authorization from a specific regulatory body that varies depending on which jurisdiction it is intended to be commercialized. The agency that regulates human medical products in the United States of America is the FDA, which divides human medical products into drugs, medical devices, combination products, and biological drugs. Synthetic tissue engineering scaffolds are usually regulated as “medical devices” when not combined with living cells or tissue-derived material. FDA categorizes medical devices into three regulatory classes (I–III) based on the level of control needed to guarantee their safety and efficacy [265]. The classification of a medical device depends on its indications for use and intended use. Also, it takes into consideration the level of risk that the device poses to the end-users. Class I includes devices with the lowest risk and Class III with the most significant risk. The class to which a medical device is assigned determines the premarketing application necessary for FDA marketing authorization [266]. Class I and II devices need a pre-market notification submission, often referred to as 510 (k), to be commercialized (CFR – Code of Federal Regulations Title 21, Chapter I, Subchapter H, Part 807 Establishment), registration and device listing for manufacturers and initial importers of devices [267]. On the other hand, a pre-market approval application (PMA) is required for class III devices (CFR – Chapter I, Subchapter H, Part 814 – pre-market approval of medical devices) [266]. The 510k notification consists of comparing the new device with a predicate medical device, whereas the PMA requires an independent demonstration of the new device’s safety and effectiveness.

*Medical device Class I, II, or III*. The easiest way to identify which category encompasses a device intended to be produced is by matching it to one of the descriptions of devices in the CFR [268]. The FDA has described more than 1700 medical devices and arranged them into 16 medical “panels” such as Dental, Orthopedic, Cardiovascular, and Radiology. These panels can be found in Parts 862 through 892 in the CFR. After choosing the panel that most relates to the medical device intended to be produced, for example, Orthopedic (Part 888), the types of devices are arranged by application, such as “Diagnostic devices”, “Prosthetic devices”, and “Surgical devices”. Each type of device has a unique “Regulation Number”, such as “888.3045 Resorbable calcium salt bone void filler device”. The regulation number represents a specific section of the regulation that describes how to identify that device, its classification (Class I, II or III), and what type of pre-market authorization is required for it (510k or PMA).

**Fig. 9:**
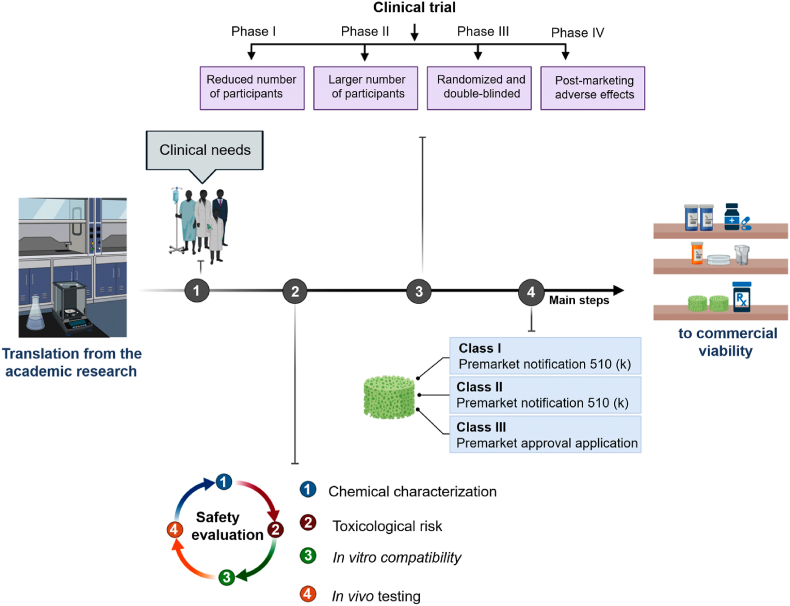
Main steps for translating from academic research to commercial viability of biomedical devices.

Once the appropriate classification is defined for the medical device intended to be produced, one can also check for a list of similar products already granted marketing authorization and access the summaries of their marketing clearances via the database [269]. This can be achieved by typing part of the device’s name and, if necessary, specify a range of approval dates. In these documents, one can find a description of the device, its intended use, technological characteristics compared with a predicate medical device, and conclusions for its substantial equivalence. The summaries of previously authorized devices provide invaluable information that bioengineers can use since the discovery stage of their products to guide them through the process of responsible product development and obtaining marketing authorization. Fig. 9 summarizes the steps for the translation from academic research to the commercial viability of biomedical devices.

## 5 Commercial landscape

Significant progress has been achieved in nanocellulose research since the discovery of cellulose nanofibrils and its first production in the early 1980s by Rayonier [270]. The number of papers published about nanocellulose has significantly increased during the last years. Since 2010, about 5000 patents refer to cellulose nanomaterials; considering CNF, approximately 70% of the patents are owned by private/for-profit companies. Companies such as Stora Enso, UPM Kymmene, and Nippon Seishi are major players, having filed more than 100 patents each in this topic. Most of the published documents are focused on production methods, surface modification, property enhancement, and applications on a laboratory scale. A significant knowledge gap exists to shift from bench development to scalable industrial production. Despite the investigations on nanocellulose during the last decades, scalable and economical material manufacturing has been a main technological bottleneck. Surprisingly, the numbers of comprehensive available techno-economic analyses on nanocellulose production are limited. In one report, the nanocellulose global market was evaluated to be around USD 65 million in 2015, with a compounded annual growth rate of approximately 30% until 2021 [271]. For example, as far as CNC production, the manufacturing cost on a large scale is estimated to be approximately USD 3632–4420 per dry ton of CNC, with the major expense related to the feedstock cost and the capital investment [271]. However, the main existing challenge in CNC industrial production is the low yield, 30%–50% depending on the pulp source. Ongoing research suggests integrating CNF and CNC production to enhance the yield and minimize cellulose loss [272]. Another challenge associated with manufacturing CNC and CNF is the high transportation cost due to the large water content in the final product relative to the dry equivalent. More research is required to retain the properties of dispersible nanocellulose after the scalable drying process to avoid the fibrils/crystals agglomeration. In addition, systematic research and techno-economic analyses are needed to address the energy and cost demand to dewater nanocellulose suspensions and to investigate the relationship between solid content and water retention. Finally, limited studies are available comparing nanocellulose-based functional structures and industrially relevant materials. Indeed, performing related benchmarking studies may remove the major barriers leading to industrial interest and to highlight the potential of related sustainable alternatives.

The first facilities to produce CNC and CNF on a commercial scale were established in 2010, and currently, more than 15 companies produce nanocellulose on large scale. Commercial CNC production (2260 ton per year on a dry basis) is currently taking place in CelluForce and Alberta Innovates in Canada, American Process and US Forest products lab, and Melodea in Israel, among other producers. The annual production of CNF on a dry basis is about 24–560 tons per year and is mainly produced by Nippon Paper and Chuetsu Pulp and Paper in Japan, the University of Maine, and American Process in the USA, and CelluComp in the UK, among others [273]. In Japan alone, over 220 companies (including Toyota Auto Body Company, Mitsui Chemical Incorporation, and Mitsubishi Motor Corporates) have engaged in nanocellulose research and development activities [274].

Cellulose is produced by approximately three trillion trees and other plants around the globe [275]. The natural abundance of cellulose offers a nearly infinite resource for the development and production of nanocellulose-based products. Sustainable materials, including nanocellulose, can address the global challenges by enhancing the performance and reducing the production costs. The increase in general awareness about the importance of sustainable and renewable resources has accelerated the commercialization of nanocellulose-based bioproducts. Some examples include deodorant sheets in adult diapers [276], self-dethatching wound dressing [277], sportswear such as GEL-KAYANO 25 shoes [278], and ink for ballpoint pen [279]. With the current progress achieved in the area of nanocellulose research and development, the number of products of industrial relevance are expected to grow.

## 6 Final remarks and perspectives

**Fig. 10:**
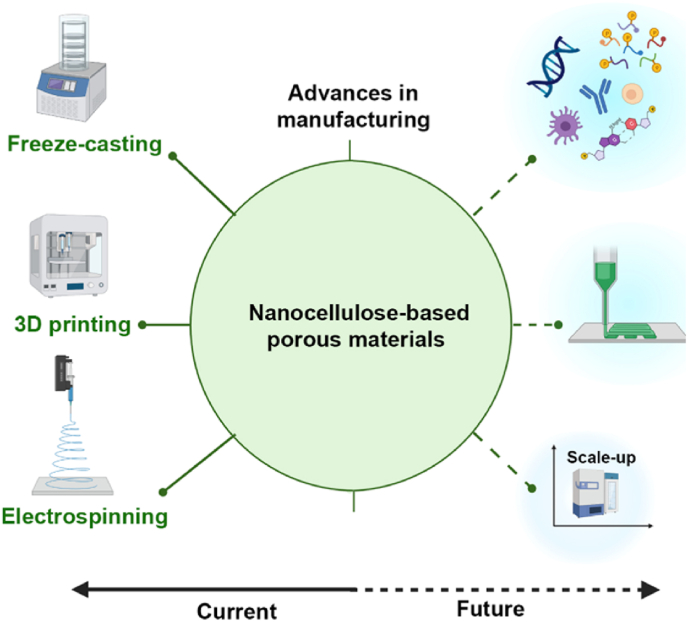
Schematic illustration of perspectives of nanocellulose-based porous materials in regenerative medicine.

Inspired by the advances in manufacturing processes and motivated by current needs, scientists and engineers have made great progress in the design of nanocellulose-based porous materials and tailoring structure-property relationships. Of particular relevance is engineering the drying process, as well as both physical and chemical cross-linking strategies. These are routes that have shown promise for developing advanced materials with good control over porosity (*e.g.*, creating macro - and mesopores) and mechanical performance (*e.g.*, Young’s modulus and stability in aqueous media) [109,280,281]. However, many challenges remain, as summarized in Fig. 10. For instance, the integration of cells (*e.g.*, stem and progenitor cells) or proteins in scaffolds of clinical relevance. Several advantages over conventional materials can be mentioned, for instance, considering the development of personalized medicine [175,282,283]. This may require post-processing (*i.e.*, a step after lyophilization), and should factor the surface energy and wettability of nanocellulose scaffolds.

Many efforts have been deployed in the area of electrospinning, and remarkable improvement (*e.g.*, solution blow spinning) have been observed in the last years [284,285]. The future perspectives for such a method are promising for developing nanocellulose-based spun fibrous mats. However, some improvements are still necessary in this field. For instance, new strategies to scale-up the production and to prepare complex structures (*i.e.*, precise spatial and temporal control) must be considered. We acknowledge the need for materials of high spatial resolution and ordered pores, *e.g.*, precision medicine, where electrospinning is not the most suitable method if compared to freeze-casting and 3D printing.

With the recent advances in 3D printing, it has been possible to combine the properties of nanocellulose to build highly ordered complex structures with tunable properties. We believe there will be an expansion in the adoption of this method promoted by the need for responsiveness, *i.e.*, 4D printing constructs [[286], [287], [288]]. Hence, nanocellulose 4D printing materials for real-life applications is a promising opportunity. Taking regenerative medicine as an example, even if still in its early stages, the technological integration of such materials holds significant potential to engineer biomimetic tissue-like constructs. To improve such constructs, the engineering of materials with the ability to self-heal after minor injuries is a fascinating prospect. In fact, as part of “mimicking natural tissues”, man-made scaffolds should spontaneously heal the defects inflicted during application, *e.g.*, pore compression, destruction of walls, *etc*. This new demand may initiate the development of nanocellulose-based smart scaffolds with self-repairing capabilities for regenerative medicine. Self-healing mechanisms have been described in hydrogels [289], and some principles have been noted as far as closure through reconnection, such as covalent bonds [290] and noncovalent interactions [291].

Finally, nanocelluloses are being already produced on large scales, in pilot and commercial facilities [292]. However, there is still a long path to successful transition of nanocellulose porous materials from the laboratory to the marketplace.

## 7 Conclusion

We discussed the major achievements in the development of nanocellulose-based micro- and nano-scale building porous materials for uses in regenerative medicine. We introduced porous materials synthesized through different approaches (*e.g.*, 3D bioprinting, freeze-casting, and electrospinning) that allow the design of materials with given structural features. The progress already achieved in this area is becoming ready to transition to a phase where knowledge translation takes a prominent role, to bring materials from the laboratory to the marketplace. The future of such materials is promising, and as the field develops, new solutions and trends will emerge. This review about the assembly of nanocellulose into versatile, high-performance materials reflects the progress in the field and the future prospects for deployment.

## Ethics approval and consent to participate

The manuscript is not related to clinical study.

## Declaration of Competing interest

The authors declare that there is no conflict of interests.

## References

[1] Caetano I.W.I.A., Ferreira F.V., dos Santos D.M., Pinheiro I.F., Lona L.M.F. (2023). Water‐dependent upcycling of eco‐friendly multifunctional nanocompartmentalized films. Adv. Sustain. Syst..

[2] Ferreira F., Pinheiro I., de Souza S., Mei L., Lona L. (2019). Polymer composites reinforced with natural fibers and nanocellulose in the automotive industry: a short review. J. Compos. Sci..

[3] Rosa R.P., Ferreira F.V., Saravia A.P.K., Rocco S.A., Sforça M.L., Gouveia R.F., Lona L.M.F. (2018). A combined computational and experimental study on the polymerization of ε-caprolactone. Ind. Eng. Chem. Res..

[4] Ferreira F.V., Trindade G.N., Lona L.M.F., Bernardes J.S., Gouveia R.F. (2019). LDPE-based composites reinforced with surface modified cellulose fibres: 3D morphological and morphometrical analyses to understand the improved mechanical performance. Eur. Polym. J..

[5] Trotter B., Ramsperger A.F.R.M., Raab P., Haberstroh J., Laforsch C. (2019). Plastic waste interferes with chemical communication in aquatic ecosystems. Sci. Rep..

[6] Lithner D., Larsson Å., Dave G. (2011). Environmental and health hazard ranking and assessment of plastic polymers based on chemical composition. Sci. Total Environ..

[7] Lebreton L.C.M., van der Zwet J., Damsteeg J.-W., Slat B., Andrady A., Reisser J. (2017). River plastic emissions to the world’s oceans. Nat. Commun..

[8] Horejs C. (2020). Solutions to plastic pollution. Nat. Rev. Mater..

[9] Geyer R., Jambeck J.R., Law K.L. (2017). Production, use, and fate of all plastics ever made. Sci. Adv..

[10] Jambeck J.R., Geyer R., Wilcox C., Siegler T.R., Perryman M., Andrady A., Narayan R., Law K.L. (2015). Plastic waste inputs from land into the ocean. Science (80-.).

[11] Jia L., Evans S., van der Linden S. (2019). Motivating actions to mitigate plastic pollution. Nat. Commun..

[12] Lebreton L., Andrady A. (2019). Future scenarios of global plastic waste generation and disposal. Palgrave Commun.

[13] Joshi C., Browning S., Seay J. (2020). Combating plastic waste via trash to tank. Nat. Rev. Earth Environ..

[14] Moradali M.F., Rehm B.H.A. (2020). Bacterial biopolymers: from pathogenesis to advanced materials. Nat. Rev. Microbiol..

[15] Rehm B.H.A. (2010). Bacterial polymers: biosynthesis, modifications and applications. Nat. Rev. Microbiol..

[16] Heinrich L.A. (2019). Future opportunities for bio-based adhesives – advantages beyond renewability. Green Chem..

[17] Santos C.C.O., Ferreira F.V., Pinheiro I.F., Lona L.M.F. (2023). Lignin valorization through polymer grafting by ring-opening polymerization and its application in health, packaging, and coating. J. Environ. Chem. Eng..

[18] Ferreira F.V., Mariano M., Lepesqueur L.S.S., Pinheiro I.F., Santos L.G., Burga-Sánchez J., Souza D.H.S., Koga-Ito C.Y., Teixeira-Neto A.A., Mei L.H.I., Gouveia R.F., Lona L.M.F. (2019). Silver nanoparticles coated with dodecanethiol used as fillers in non-cytotoxic and antifungal PBAT surface based on nanocomposites. Mater. Sci. Eng. C..

[19] Ferreira F.V., Cividanes L.S., Gouveia R.F., Lona L.M. (2019). An overview on properties and applications of poly(butylene adipate- co -terephthalate)-PBAT based composites. Polym. Eng. Sci..

[20] Ajdary R., Tardy B.L., Mattos B.D., Bai L., Rojas O.J. (2020). Plant nanomaterials and inspiration from nature: water interactions and hierarchically structured hydrogels. Adv. Mater..

[21] Thomas B., Raj M.C., B A.K., H R.M., Joy J., Moores A., Drisko G.L., Sanchez C. (2018). Nanocellulose, a versatile green platform: from biosources to materials and their applications. Chem. Rev..

[22] Khine Y.Y., Stenzel M.H. (2020). Surface modified cellulose nanomaterials: a source of non-spherical nanoparticles for drug delivery. Mater. Horizons..

[23] Tu H., Zhu M., Duan B., Zhang L. (2020). Recent progress in high‐strength and robust regenerated cellulose materials. Adv. Mater..

[24] Kobayashi K., Ura Y., Kimura S., Sugiyama J. (2018). Outstanding toughness of Cherry Bark achieved by helical spring structure of rigid cellulose fiber combined with flexible layers of lipid polymers. Adv. Mater..

[25] Pinheiro I.F., Ferreira F.V., Souza D.H.S., Gouveia R.F., Lona L.M.F., Morales A.R., Mei L.H.I. (2017). Mechanical, rheological and degradation properties of PBAT nanocomposites reinforced by functionalized cellulose nanocrystals. Eur. Polym. J..

[26] Pinheiro I.F., Ferreira F.V., Alves G.F., Rodolfo A., Morales A.R., Mei L.H.I. (2019). Biodegradable PBAT-based nanocomposites reinforced with functionalized cellulose nanocrystals from pseudobombax munguba: rheological, thermal, mechanical and biodegradability properties. J. Polym. Environ..

[27] Ferreira F.V., Pinheiro I.F., Mariano M., Cividanes L.S., Costa J.C.M., Nascimento N.R., Kimura S.P.R., Neto J.C.M., Lona L.M.F. (2019). Environmentally friendly polymer composites based on PBAT reinforced with natural fibers from the amazon forest. Polym. Compos..

[28] Kontturi E., Laaksonen P., Linder M.B., Nonappa, Gröschel A.H., Rojas O.J., Ikkala O. (2018). Advanced materials through assembly of nanocelluloses. Adv. Mater..

[29] Klemm D., Heublein B., Fink H.P., Bohn A. (2005). Cellulose: fascinating biopolymer and sustainable raw material. Angew. Chemie - Int. Ed..

[30] Ferreira F.V., Mariano M., Rabelo S.C., Gouveia R.F., Lona L.M.F. (2018). Isolation and surface modification of cellulose nanocrystals from sugarcane bagasse waste: from a micro- to a nano-scale view. Appl. Surf. Sci..

[31] Moon R.J., Martini A., Nairn J., Simonsen J., Youngblood J. (2011). Cellulose nanomaterials review: structure, properties and nanocomposites. Chem. Soc. Rev..

[32] Rol F., Belgacem M.N., Gandini A., Bras J. (2019). Recent advances in surface-modified cellulose nanofibrils. Prog. Polym. Sci..

[33] Du H., Parit M., Wu M., Che X., Wang Y., Zhang M., Wang R., Zhang X., Jiang Z., Li B. (2020). Sustainable valorization of paper mill sludge into cellulose nanofibrils and cellulose nanopaper. J. Hazard Mater..

[34] Ferreira F.V., Dufresne A., Pinheiro I.F., Souza D.H.S., Gouveia R.F., Mei L.H.I., Lona L.M.F. (2018). How do cellulose nanocrystals affect the overall properties of biodegradable polymer nanocomposites: a comprehensive review. Eur. Polym. J..

[35] Salas C., Nypelö T., Rodriguez-Abreu C., Carrillo C., Rojas O.J. (2014). Nanocellulose properties and applications in colloids and interfaces. Curr. Opin. Colloid Interface Sci..

[36] Mittal N., Benselfelt T., Ansari F., Gordeyeva K., Roth S.V., Wågberg L., Söderberg L.D. (2019). Ion‐specific assembly of strong, tough, and stiff biofibers. Angew. Chemie Int. Ed..

[37] Ferreira F.V., Pinheiro I.F., Gouveia R.F., Thim G.P., Lona L.M.F. (2018). Functionalized cellulose nanocrystals as reinforcement in biodegradable polymer nanocomposites. Polym. Compos..

[38] Ferreira F.V., Mariano M., Pinheiro I.F., Cazalini E.M., Souza D.H.S., Lepesqueur L.S.S., Koga-Ito C.Y., Gouveia R.F., Lona L.M.F. (2019). Cellulose nanocrystal‐based poly(butylene adipate‐co‐terephthalate) nanocomposites covered with antimicrobial silver thin films. Polym. Eng. Sci..

[39] De France K., Zeng Z., Wu T., Nyström G. (2020). Functional materials from nanocellulose: utilizing structure–property relationships in bottom‐up fabrication. Adv. Mater..

[40] Niinivaara E., Cranston E.D. (2020). Bottom-up assembly of nanocellulose structures. Carbohydr. Polym..

[41] Lamm M.E., Li K., Qian J., Wang L., Lavoine N., Newman R., Gardner D.J., Li T., Hu L., Ragauskas A.J., Tekinalp H., Kunc V., Ozcan S. (2021). Recent advances in functional materials through cellulose nanofiber templating. Adv. Mater..

[42] Raza M., Abu-Jdayil B. (2022). Cellulose nanocrystals from lignocellulosic feedstock: a review of production technology and surface chemistry modification. Cellulose.

[43] Thomas P., Duolikun T., Rumjit N.P., Moosavi S., Lai C.W., Bin Johan M.R., Fen L.B. (2020). Comprehensive review on nanocellulose: recent developments, challenges and future prospects. J. Mech. Behav. Biomed. Mater..

[44] Tran A., Boott C.E., MacLachlan M.J. (2020). Understanding the self‐assembly of cellulose nanocrystals—toward Chiral photonic materials. Adv. Mater..

[45] Noremylia M.B., Hassan M.Z., Ismail Z. (2022). Recent advancement in isolation, processing, characterization and applications of emerging nanocellulose: a review. Int. J. Biol. Macromol..

[46] Zhu M., Sun Z., Zhang Z., Shi Q., He T., Liu H., Chen T., Lee C. (2020). Haptic-feedback smart glove as a creative human-machine interface (HMI) for virtual/augmented reality applications. Sci. Adv..

[47] Wågberg L., Erlandsson J. (2020). The use of layer‐by‐layer self‐assembly and nanocellulose to prepare advanced functional materials. Adv. Mater..

[48] Tardy B.L., Mattos B.D., Otoni C.G., Beaumont M., Majoinen J., Kämäräinen T., Rojas O.J. (2021). Deconstruction and reassembly of renewable polymers and biocolloids into next generation structured materials. Chem. Rev..

[49] Lv P., Lu X., Wang L., Feng W. (2021). Nanocellulose‐based functional materials: from Chiral photonics to soft actuator and energy storage. Adv. Funct. Mater..

[50] Klemm D., Kramer F., Moritz S., Lindström T., Ankerfors M., Gray D., Dorris A. (2011). Nanocelluloses: a new family of nature-based materials. Angew. Chemie Int. Ed..

[51] Lin N., Huang J., Dufresne A. (2012). Preparation, properties and applications of polysaccharide nanocrystals in advanced functional nanomaterials: a review. Nanoscale.

[52] Novo L.P., Bras J., García A., Belgacem N., Curvelo A.A.S. (2015). Subcritical water: a method for green production of cellulose nanocrystals. ACS Sustain. Chem. Eng..

[53] Pereira B., Arantes V. (2020). Production of cellulose nanocrystals integrated into a biochemical sugar platform process via enzymatic hydrolysis at high solid loading. Ind. Crops Prod..

[54] Zhang R., Liu Y. (2018). High energy oxidation and organosolv solubilization for high yield isolation of cellulose nanocrystals (CNC) from Eucalyptus hardwood. Sci. Rep..

[55] Sacui I.A., Nieuwendaal R.C., Burnett D.J., Stranick S.J., Jorfi M., Weder C., Foster E.J., Olsson R.T., Gilman J.W. (2014). Comparison of the properties of cellulose nanocrystals and cellulose nanofibrils isolated from bacteria, tunicate, and wood processed using acid, enzymatic, mechanical, and oxidative methods. ACS Appl. Mater. Interfaces.

[56] Kassab Z., Kassem I., Hannache H., Bouhfid R., Qaiss A.E.K., El Achaby M. (2020). Tomato plant residue as new renewable source for cellulose production: extraction of cellulose nanocrystals with different surface functionalities. Cellulose.

[57] Ferreira F.V., Pinheiro I.F., Gouveia R.F., Thim G.P., Lona L.M.F. (2017). Functionalized cellulose nanocrystals as reinforcement in biodegradable polymer nanocomposites. Polym. Compos..

[58] Hasani M., Cranston E.D., Westman G., Gray D.G. (2008). Cationic surface functionalization of cellulose nanocrystals. Soft Matter.

[59] Rosa R.P., Ferreira F.V., dos Santos D.M., Lona L.M.F. (2022). Cellulose nanocrystals as initiator of ring-opening polymerization of ε-caprolactone: mathematical modeling and experimental verification. Eur. Polym. J..

[60] Saito T., Isogai A. (2004). TEMPO-mediated oxidation of native cellulose. The effect of oxidation conditions on chemical and crystal structures of the water-Insoluble fractions. Biomacromolecules.

[61] Eyley S., Thielemans W. (2014). Surface modification of cellulose nanocrystals. Nanoscale.

[62] Cherian R.M., Tharayil A., Varghese R.T., Antony T., Kargarzadeh H., Chirayil C.J., Thomas S. (2022). A review on the emerging applications of nano-cellulose as advanced coatings. Carbohydr. Polym..

[63] Silva F.A.G.S., Dourado F., Gama M., Poças F. (2020). Nanocellulose bio-based composites for food packaging. Nanomaterials.

[64] Trache D., Hussin M.H., Haafiz M.K.M., Thakur V.K. (2017). Recent progress in cellulose nanocrystals: sources and production. Nanoscale.

[65] Isogai A. (2020). Emerging nanocellulose technologies: recent developments. Adv. Mater..

[66] Tao H., Lavoine N., Jiang F., Tang J., Lin N. (2020). Reducing end modification on cellulose nanocrystals: strategy, characterization, applications and challenges. Nanoscale Horiz..

[67] Isogai A., Hänninen T., Fujisawa S., Saito T. (2018). Review: catalytic oxidation of cellulose with nitroxyl radicals under aqueous conditions. Prog. Polym. Sci..

[68] Šturcova A., His I., Apperley D.C., Sugiyama J., Jarvis M.C. (2004). Structural details of crystalline cellulose from higher plants. Biomacromolecules.

[69] Camarero Espinosa S., Kuhnt T., Foster E.J., Weder C. (2013). Isolation of thermally stable cellulose nanocrystals by phosphoric acid hydrolysis. Biomacromolecules.

[70] Cherpak V., Korolovych V.F., Geryak R., Turiv T., Nepal D., Kelly J., Bunning T.J., Lavrentovich O.D., Heller W.T., Tsukruk V.V. (2018). Robust Chiral organization of cellulose nanocrystals in Capillary Confinement. Nano Lett..

[71] Chan C.L.C., Bay M.M., Jacucci G., Vadrucci R., Williams C.A., de Kerkhof G.T., Parker R.M., Vynck K., Frka‐Petesic B., Vignolini S. (2019). Visual appearance of Chiral nematic cellulose‐based photonic films: angular and polarization independent color response with a twist. Adv. Mater..

[72] Dumanli A.G., Kamita G., Landman J., van der Kooij H., Glover B.J., Baumberg J.J., Steiner U., Vignolini S. (2014). Controlled, bio‐inspired self‐assembly of cellulose‐based Chiral reflectors. Adv. Opt. Mater..

[73] Frka‐Petesic B., Kelly J.A., Jacucci G., Guidetti G., Kamita G., Crossette N.P., Hamad W.Y., MacLachlan M.J., Vignolini S. (2020). Retrieving the Coassembly pathway of composite cellulose nanocrystal photonic films from their angular optical response. Adv. Mater..

[74] Heise K., Koso T., King A.W.T., Nypelö T., Penttilä P., Tardy B.L., Beaumont M. (2022). Spatioselective surface chemistry for the production of functional and chemically anisotropic nanocellulose colloids. J. Mater. Chem. A..

[75] Klockars K.W., Greca L.G., Majoinen J., Mihhels K., Rojas O.J., Tardy B.L. (2023). Drying stresses in cellulose nanocrystal coatings: impact of molecular and macromolecular additives. Carbohydr. Polym..

[76] Borrero-López A.M., Greca L.G., Rojas O.J., Tardy B.L. (2023). Controlling superstructure formation and macro-scale adhesion via confined evaporation of cellulose nanocrystals. Cellulose.

[77] Ariga K., Jia X., Song J., Hill J.P., Leong D.T., Jia Y., Li J. (2020). Nanoarchitektonik als ein Ansatz zur Erzeugung bioähnlicher hierarchischer Organisate. Angew. Chemie.

[78] Liu Y.-T., Zhang P., Sun N., Anasori B., Zhu Q.-Z., Liu H., Gogotsi Y., Xu B. (2018). Self-assembly of transition metal oxide nanostructures on MXene nanosheets for fast and stable lithium storage. Adv. Mater..

[79] Grzelczak M., Liz-Marzán L.M., Klajn R. (2019). Stimuli-responsive self-assembly of nanoparticles. Chem. Soc. Rev..

[80] Wang L., Urbas A.M., Li Q. (2020). Nature‐inspired emerging Chiral liquid crystal nanostructures: from molecular self‐assembly to DNA mesophase and nanocolloids. Adv. Mater..

[81] Lei S., Huang D., Liu S., Chen M., Ma R., Zeng M., Li D., Ma W., Wang L., Cheng Z. (2021). Templating synthesis of natural cotton-based hierarchically structured carbon hollow microfibers for high-performance solar vapor generation. J. Mater. Chem. A..

[82] Liu J., Zhang Y., Li S., Valenzuela C., Shi S., Jiang C., Wu S., Ye L., Wang L., Zhou Z. (2023). Emerging materials and engineering strategies for performance advance of radiative sky cooling technology. Chem. Eng. J..

[83] Lv P., Yang X., Bisoyi H.K., Zeng H., Zhang X., Chen Y., Xue P., Shi S., Priimagi A., Wang L., Feng W., Li Q. (2021). Stimulus-driven liquid metal and liquid crystal network actuators for programmable soft robotics, Mater. Horizons.

[84] Yang J., Zhang X., Zhang X., Wang L., Feng W., Li Q. (2021). Beyond the visible: bioinspired infrared adaptive materials. Adv. Mater..

[85] Qi Y., Guo Y., Liza A.A., Yang G., Sipponen M.H., Guo J., Li H. (2023). Nanocellulose: a review on preparation routes and applications in functional materials. Cellulose.

[86] Hao X., Lv Z., Wang H., Rao J., Liu Q., Lü B., Peng F. (2022). Top-down production of sustainable and scalable hemicellulose nanocrystals. Biomacromolecules.

[87] Abbasi Moud A. (2022). Advanced cellulose nanocrystals (CNC) and cellulose nanofibrils (CNF) aerogels: bottom-up assembly perspective for production of adsorbents. Int. J. Biol. Macromol..

[88] Lizundia E., Puglia D., Nguyen T.D., Armentano I. (2020). Cellulose nanocrystal based multifunctional nanohybrids. Prog. Mater. Sci..

[89] Wang Q., Yao Q., Liu J., Sun J., Zhu Q., Chen H. (2019). Processing nanocellulose to bulk materials: a review. Cellulose.

[90] Ostrovidov S., Salehi S., Costantini M., Suthiwanich K., Ebrahimi M., Sadeghian R.B., Fujie T., Shi X., Cannata S., Gargioli C., Tamayol A., Dokmeci M.R., Orive G., Swieszkowski W., Khademhosseini A. (2019). 3D bioprinting in skeletal muscle tissue engineering. Small.

[91] Prendergast M.E., Burdick J.A. (2020). Recent advances in enabling technologies in 3D printing for precision medicine. Adv. Mater..

[92] Ashammakhi N., Ahadian S., Xu C., Montazerian H., Ko H., Nasiri R., Barros N., Khademhosseini A. (2019). Bioinks and bioprinting technologies to make heterogeneous and biomimetic tissue constructs. Mater. Today Bio..

[93] Chatterjee K., Ghosh T.K. (2020). 3D printing of textiles: potential roadmap to printing with fibers. Adv. Mater..

[94] Bedell M.L., Navara A.M., Du Y., Zhang S., Mikos A.G. (2020). Polymeric systems for bioprinting. Chem. Rev..

[95] Huan S., Ajdary R., Bai L., Klar V., Rojas O.J. (2019). Low solids emulsion gels based on nanocellulose for 3D-printing. Biomacromolecules.

[96] Malda J., Visser J., Melchels F.P., Jüngst T., Hennink W.E., Dhert W.J.A., Groll J., Hutmacher D.W. (2013). 25th anniversary article: engineering hydrogels for biofabrication. Adv. Mater..

[97] Håkansson K.M.O., Henriksson I.C., de la Peña Vázquez C., Kuzmenko V., Markstedt K., Enoksson P., Gatenholm P. (2016). Solidification of 3D printed nanofibril hydrogels into functional 3D cellulose structures. Adv. Mater. Technol..

[98] Hausmann M.K., Rühs P.A., Siqueira G., Läuger J., Libanori R., Zimmermann T., Studart A.R. (2018). Dynamics of cellulose nanocrystal alignment during 3D printing. ACS Nano.

[99] Truby R.L., Lewis J.A. (2016). Printing soft matter in three dimensions. Nature.

[100] Wang J., Chiappone A., Roppolo I., Shao F., Fantino E., Lorusso M., Rentsch D., Dietliker K., Pirri C.F., Grutzmacher H. (2018). All-in-One cellulose nanocrystals for 3D printing of nanocomposite hydrogels. Angew. Chemie Int. Ed..

[101] Studart A.R. (2016). Additive manufacturing of biologically-inspired materials. Chem. Soc. Rev..

[102] Siqueira G., Kokkinis D., Libanori R., Hausmann M.K., Gladman A.S., Neels A., Tingaut P., Zimmermann T., Lewis J.A., Studart A.R. (2017). Cellulose nanocrystal inks for 3D printing of textured cellular architectures. Adv. Funct. Mater..

[103] Rees A., Powell L.C., Chinga-Carrasco G., Gethin D.T., Syverud K., Hill K.E., Thomas D.W. (2015). 3D bioprinting of carboxymethylated-periodate oxidized nanocellulose constructs for wound dressing applications. BioMed Res. Int..

[104] Gungor-Ozkerim P.S., Inci I., Zhang Y.S., Khademhosseini A., Dokmeci M.R. (2018). Bioinks for 3D bioprinting: an overview. Biomater. Sci..

[105] Xin S., Chimene D., Garza J.E., Gaharwar A.K., Alge D.L. (2019). Clickable PEG hydrogel microspheres as building blocks for 3D bioprinting. Biomater. Sci..

[106] Fourmann O., Hausmann M.K., Neels A., Schubert M., Nyström G., Zimmermann T., Siqueira G. (2021). 3D printing of shape-morphing and antibacterial anisotropic nanocellulose hydrogels. Carbohydr. Polym..

[107] Svensson A., Larsson P.T., Salazar-Alvarez G., Wågberg L. (2013). Preparation of dry ultra-porous cellulosic fibres: characterization and possible initial uses. Carbohydr. Polym..

[108] Françon H., Wang Z., Marais A., Mystek K., Piper A., Granberg H., Malti A., Gatenholm P., Larsson P.A., Wågberg L. (2020). Ambient‐dried, 3D‐printable and electrically conducting cellulose nanofiber aerogels by inclusion of functional polymers. Adv. Funct. Mater..

[109] Wu T., Zeng Z., Siqueira G., De France K., Sivaraman D., Schreiner C., Figi R., Zhang Q., Nyström G. (2020). Dual-porous cellulose nanofibril aerogels via modular drying and cross-linking. Nanoscale.

[110] Saito T., Kimura S., Nishiyama Y., Isogai A. (2007). Cellulose nanofibers prepared by TEMPO-mediated oxidation of native cellulose. Biomacromolecules.

[111] Weishaupt R., Siqueira G., Schubert M., Tingaut P., Maniura-Weber K., Zimmermann T., Thöny-Meyer L., Faccio G., Ihssen J. (2015). TEMPO-oxidized nanofibrillated cellulose as a high density carrier for bioactive molecules. Biomacromolecules.

[112] Markstedt K., Mantas A., Tournier I., Martínez Ávila H., Hägg D., Gatenholm P. (2015). 3D bioprinting human chondrocytes with nanocellulose–alginate bioink for cartilage tissue engineering applications. Biomacromolecules.

[113] Valot L., Martinez J., Mehdi A., Subra G. (2019). Chemical insights into bioinks for 3D printing. Chem. Soc. Rev..

[114] Ajdary R., Ezazi N.Z., Correia A., Kemell M., Huan S., Ruskoaho H.J., Hirvonen J., Santos H.A., Rojas O.J. (2020). Multifunctional 3D‐printed patches for long‐term drug release therapies after myocardial infarction. Adv. Funct. Mater..

[115] Hamedi M.M., Hajian A., Fall A.B., Håkansson K., Salajkova M., Lundell F., Wågberg L., Berglund L.A. (2014). Highly conducting, strong nanocomposites based on nanocellulose-assisted aqueous dispersions of single-wall carbon nanotubes. ACS Nano.

[116] Wang M., Anoshkin I.V., Nasibulin A.G., Korhonen J.T., Seitsonen J., Pere J., Kauppinen E.I., Ras R.H.A., Ikkala O. (2013). Modifying native nanocellulose aerogels with carbon nanotubes for mechanoresponsive conductivity and pressure sensing. Adv. Mater..

[117] Gao K., Shao Z., Li J., Wang X., Peng X., Wang W., Wang F. (2013). Cellulose nanofiber–graphene all solid-state flexible supercapacitors. J. Mater. Chem. A..

[118] Zhang K., Ketterle L., Järvinen T., Lorite G.S., Hong S., Liimatainen H. (2020). Self-assembly of graphene oxide and cellulose nanocrystals into continuous filament via interfacial nanoparticle complexation. Mater. Des..

[119] Olsson R.T., Azizi Samir M.A.S., Salazar-Alvarez G., Belova L., Ström V., Berglund L.A., Ikkala O., Nogués J., Gedde U.W. (2010). Making flexible magnetic aerogels and stiff magnetic nanopaper using cellulose nanofibrils as templates. Nat. Nanotechnol..

[120] Blaeser A., Duarte Campos D.F., Puster U., Richtering W., Stevens M.M., Fischer H. (2016). Controlling shear stress in 3D bioprinting is a key factor to balance printing resolution and stem cell integrity. Adv. Healthc. Mater..

[121] Sydney Gladman A., Matsumoto E.A., Nuzzo R.G., Mahadevan L., Lewis J.A. (2016). Biomimetic 4D printing. Nat. Mater..

[122] Osorio D.A., Lee B.E.J., Kwiecien J.M., Wang X., Shahid I., Hurley A.L., Cranston E.D., Grandfield K. (2019). Cross-linked cellulose nanocrystal aerogels as viable bone tissue scaffolds. Acta Biomater..

[123] Shao G., Hanaor D.A.H., Shen X., Gurlo A. (2020). Freeze casting: from low‐dimensional building blocks to aligned porous structures—a review of novel materials, methods, and applications. Adv. Mater..

[124] Bai H., Chen Y., Delattre B., Tomsia A.P., Ritchie R.O. (2015). Bioinspired large-scale aligned porous materials assembled with dual temperature gradients. Sci. Adv..

[125] Chau M., De France K.J., Kopera B., Machado V.R., Rosenfeldt S., Reyes L., Chan K.J.W., Förster S., Cranston E.D., Hoare T., Kumacheva E. (2016). Composite hydrogels with tunable anisotropic morphologies and mechanical properties. Chem. Mater..

[126] Otoni C.G., Figueiredo J.S.L., Capeletti L.B., Cardoso M.B., Bernardes J.S., Loh W. (2019). Tailoring the antimicrobial response of cationic nanocellulose-based foams through cryo-templating. ACS Appl. Bio Mater..

[127] Deville S. (2006). Freezing as a path to build complex composites. Science (80-.).

[128] Mariano M., Bernardes J. da S., Strauss M. (2018). Mold heat conductance as drive force for tuning freeze-casted nanocellulose foams microarchitecture. Mater. Lett..

[129] Lavoine N., Bergström L. (2017). Nanocellulose-based foams and aerogels: processing, properties, and applications. J. Mater. Chem. A..

[130] Ferreira E.S., Cranston E.D., Rezende C.A. (2020). Naturally hydrophobic foams from lignocellulosic fibers prepared by oven-drying. ACS Sustain. Chem. Eng..

[131] Faruk O., Bledzki A.K., Matuana L.M. (2007). Microcellular foamed wood-plastic composites by different processes: a review. Macromol. Mater. Eng..

[132] Munier P., Gordeyeva K., Bergström L., Fall A.B. (2016). Directional freezing of nanocellulose dispersions aligns the rod-like particles and produces low-density and robust particle networks. Biomacromolecules.

[133] Nechyporchuk O., Belgacem M.N., Pignon F. (2016). Current progress in rheology of cellulose nanofibril suspensions. Biomacromolecules.

[134] Kuang Y., Chen C., He S., Hitz E.M., Wang Y., Gan W., Mi R., Hu L. (2019). A high‐performance self‐regenerating solar evaporator for continuous water desalination. Adv. Mater..

[135] Thorkelsson K., Bai P., Xu T. (2015). Self-assembly and applications of anisotropic nanomaterials: a review. Nano Today.

[136] Korhonen O., Budtova T. (2020). All-cellulose composite aerogels and cryogels. Compos. Part A Appl. Sci. Manuf..

[137] Li L., Tao H., Wu B., Zhu G., Li K., Lin N. (2018). Triazole end-grafting on cellulose nanocrystals for water-redispersion improvement and reactive enhancement to nanocomposites. ACS Sustain. Chem. Eng..

[138] Courtenay J.C., Filgueiras J.G., DeAzevedo E.R., Jin Y., Edler K.J., Sharma R.I., Scott J.L. (2019). Mechanically robust cationic cellulose nanofibril 3D scaffolds with tuneable biomimetic porosity for cell culture. J. Mater. Chem. B.

[139] Mercante L.A., Scagion V.P., Migliorini F.L., Mattoso L.H.C., Correa D.S. (2017). Electrospinning-based (bio)sensors for food and agricultural applications: a review. TrAC Trends Anal. Chem..

[140] Baji A., Mai Y.-W., Wong S.-C., Abtahi M., Chen P. (2010). Electrospinning of polymer nanofibers: effects on oriented morphology, structures and tensile properties. Compos. Sci. Technol..

[141] Zhang J., Liu L., Si Y., Yu J., Ding B. (2020). Electrospun nanofibrous membranes: an effective arsenal for the purification of emulsified oily wastewater. Adv. Funct. Mater..

[142] Xue J., Xie J., Liu W., Xia Y. (2017). Electrospun nanofibers: new concepts, materials, and applications. Acc. Chem. Res..

[143] Chew S.Y., Wen J., Yim E.K.F., Leong K.W. (2005). Sustained release of proteins from electrospun biodegradable fibers. Biomacromolecules.

[144] Deepthi S., Nivedhitha Sundaram M., Deepti Kadavan J., Jayakumar R. (2016). Layered chitosan-collagen hydrogel/aligned PLLA nanofiber construct for flexor tendon regeneration. Carbohydr. Polym..

[145] Xie J., MacEwan M.R., Ray W.Z., Liu W., Siewe D.Y., Xia Y. (2010). Radially aligned, electrospun nanofibers as dural Substitutes for wound closure and tissue regeneration applications. ACS Nano.

[146] Li D., Wang Y., Xia Y. (2003). Electrospinning of polymeric and ceramic nanofibers as uniaxially aligned arrays. Nano Lett..

[147] Liu Y., Zhang X., Xia Y., Yang H. (2010). Magnetic-field-assisted electrospinning of aligned straight and wavy polymeric nanofibers. Adv. Mater..

[148] Yang D., Lu B., Zhao Y., Jiang X. (2007). Fabrication of aligned fibrous arrays by magnetic electrospinning. Adv. Mater..

[149] Liu W., Thomopoulos S., Xia Y. (2012). Electrospun nanofibers for regenerative medicine. Adv. Healthc. Mater..

[150] Li D., Ouyang G., McCann J.T., Xia Y. (2005). Collecting electrospun nanofibers with patterned electrodes. Nano Lett..

[151] Li K., Wang J., Liu X., Xiong X., Liu H. (2012). Biomimetic growth of hydroxyapatite on phosphorylated electrospun cellulose nanofibers. Carbohydr. Polym..

[152] Joshi M.K., Pant H.R., Tiwari A.P., Maharjan B., Liao N., Kim H.J., Park C.H., Kim C.S. (2016). Three-dimensional cellulose sponge: fabrication, characterization, biomimetic mineralization, and in vitro cell infiltration. Carbohydr. Polym..

[153] Shi Q., Zhou C., Yue Y., Guo W., Wu Y., Wu Q. (2012). Mechanical properties and in vitro degradation of electrospun bio-nanocomposite mats from PLA and cellulose nanocrystals. Carbohydr. Polym..

[154] Ago M., Okajima K., Jakes J.E., Park S., Rojas O.J. (2012). Lignin-based electrospun nanofibers reinforced with cellulose nanocrystals. Biomacromolecules.

[155] Zhou C., Shi Q., Guo W., Terrell L., Qureshi A.T., Hayes D.J., Wu Q. (2013). Electrospun bio-nanocomposite scaffolds for bone tissue engineering by cellulose nanocrystals reinforcing maleic anhydride grafted PLA. ACS Appl. Mater. Interfaces.

[156] Reneker D.H., Yarin A.L. (2008). Electrospinning jets and polymer nanofibers. Polymer (Guildf).

[157] Favier V., Dendievel R., Canova G., Cavaille J.Y., Gilormini P. (1997). Simulation and modeling of three-dimensional percolating structures: case of a latex matrix reinforced by a network of cellulose fibers. Acta Mater..

[158] Mohammadi P., Toivonen M.S., Ikkala O., Wagermaier W., Linder M.B. (2017). Aligning cellulose nanofibril dispersions for tougher fibers. Sci. Rep..

[159] Yang F., Murugan R., Wang S., Ramakrishna S. (2005). Electrospinning of nano/micro scale poly(l-lactic acid) aligned fibers and their potential in neural tissue engineering. Biomaterials.

[160] Xie J., Willerth S.M., Li X., Macewan M.R., Rader A., Sakiyama-Elbert S.E., Xia Y. (2009). The differentiation of embryonic stem cells seeded on electrospun nanofibers into neural lineages. Biomaterials.

[161] Yin Z., Chen X., Chen J.L., Shen W.L., Hieu Nguyen T.M., Gao L., Ouyang H.W. (2010). The regulation of tendon stem cell differentiation by the alignment of nanofibers. Biomaterials.

[162] Xie J., MacEwan M.R., Schwartz A.G., Xia Y. (2010). Electrospun nanofibers for neural tissue engineering. Nanoscale.

[163] Zong X., Bien H., Chung C., Yin L., Fang D., Hsiao B., Chu B., Entcheva E. (2005). Electrospun fine-textured scaffolds for heart tissue constructs. Biomaterials.

[164] Lamarra J., Calienni M.N., Rivero S., Pinotti A. (2020). Electrospun nanofibers of poly(vinyl alcohol) and chitosan-based emulsions functionalized with cabreuva essential oil. Int. J. Biol. Macromol..

[165] Jacobs J., Pavlović D., Prydderch H., Moradi M.-A., Ibarboure E., Heuts J.P.A., Lecommandoux S., Heise A. (2019). Polypeptide nanoparticles obtained from emulsion polymerization of amino acid N-carboxyanhydrides. J. Am. Chem. Soc..

[166] Hu X., Liu S., Zhou G., Huang Y., Xie Z., Jing X. (2014). Electrospinning of polymeric nanofibers for drug delivery applications. J. Control. Release.

[167] Li W., Garmendia N., Pérez de Larraya U., Ding Y., Detsch R., Grünewald A., Roether J.A., Schubert D.W., Boccaccini A.R. (2014). 45S5 bioactive glass-based scaffolds coated with cellulose nanowhiskers for bone tissue engineering. RSC Adv..

[168] Chen Q., Garcia R.P., Munoz J., Pérez de Larraya U., Garmendia N., Yao Q., Boccaccini A.R. (2015). Cellulose nanocrystals—bioactive glass hybrid coating as bone substitutes by electrophoretic co-deposition: in situ control of mineralization of bioactive glass and enhancement of osteoblastic performance. ACS Appl. Mater. Interfaces.

[169] Huang Y., Song J., Yang C., Long Y., Wu H. (2019). Scalable manufacturing and applications of nanofibers, Mater. Today Off..

[170] Szczęśniak B., Borysiuk S., Choma J., Jaroniec M. (2020). Mechanochemical synthesis of highly porous materials. Mater. Horizons.

[171] Garemark J., Yang X., Sheng X., Cheung O., Sun L., Berglund L.A., Li Y. (2020). Top-down approach making anisotropic cellulose aerogels as universal substrates for multifunctionalization. ACS Nano.

[172] Vernengo A.J., Grad S., Eglin D., Alini M., Li Z. (2020). Bioprinting tissue analogues with decellularized extracellular matrix bioink for regeneration and tissue models of cartilage and intervertebral discs. Adv. Funct. Mater..

[173] Ferreira F.V., Otoni C.G., De France K.J., Barud H.S., Lona L.M.F., Cranston E.D., Rojas O.J. (2020). Porous nanocellulose gels and foams: breakthrough status in the development of scaffolds for tissue engineering. Mater. Today.

[174] Liu W., Du H., Zhang M., Liu K., Liu H., Xie H., Zhang X., Si C. (2020). Bacterial cellulose-based composite scaffolds for biomedical applications: a review. ACS Sustain. Chem. Eng..

[175] Clegg J.R., Wagner A.M., Shin S.R., Hassan S., Khademhosseini A., Peppas N.A. (2019). Modular fabrication of intelligent material-tissue interfaces for bioinspired and biomimetic devices. Prog. Mater. Sci..

[176] Souza L., Ferreira F.V., Lopes J.H., Camilli J.A., Martin R.A. (2022). Cancer inhibition and in vivo osteointegration and compatibility of gallium-doped bioactive glasses for osteosarcoma applications. ACS Appl. Mater. Interfaces.

[177] Lopes J.H., Souza L.P., Domingues J.A., Ferreira F.V., Alencar Hausen M., Camilli J.A., Martin R.A., Rezende Duek E.A., Mazali I.O., Bertran C.A. (2019). In vitro and in vivo osteogenic potential of niobium‐doped 45S5 bioactive glass: a comparative study. J. Biomed. Mater. Res. Part B Appl. Biomater..

[178] Medeiros G.S., Oliveira L.F.M., Ferreira F.V., Souza L.P., Martin R.A., de Oliveira I.R., Lopes J.H. (2023). A perfect pair: niobium- and gallium-doped ceramic biomaterial enabled by coupled synthesis method with potential application for bone regeneration and cancer-targeted therapy. J. Non-Cryst. Solids.

[179] Souza L.P.L., Lopes J.H., Ferreira F.V., Martin R.A., Bertran C.A., Camilli J.A. (2019). Evaluation of effectiveness of 45S5 bioglass doped with niobium for repairing critical‐sized bone defect in in vitro and in vivo models. J. Biomed. Mater. Res. Part A..

[180] Li W., Liu J., Zhao D. (2016). Mesoporous materials for energy conversion and storage devices. Nat. Rev. Mater..

[181] Oliviero O., Ventre M., Netti P.A. (2012). Functional porous hydrogels to study angiogenesis under the effect of controlled release of vascular endothelial growth factor. Acta Biomater..

[182] Du H., Parit M., Liu K., Zhang M., Jiang Z., Huang T.-S., Zhang X., Si C. (2021). Multifunctional cellulose nanopaper with superior water-resistant, conductive, and antibacterial properties functionalized with chitosan and polypyrrole. ACS Appl. Mater. Interfaces.

[183] Hu Z., Marway H.S., Kasem H., Pelton R., Cranston E.D. (2016). Dried and redispersible cellulose nanocrystal pickering emulsions. ACS Macro Lett..

[184] Kim Y., Kim Y.K., Kim S., Harbottle D., Lee J.W. (2017). Nanostructured potassium copper hexacyanoferrate-cellulose hydrogel for selective and rapid cesium adsorption. Chem. Eng. J..

[185] Zhang W., Zhang Y., Lu C., Deng Y. (2012). Aerogels from crosslinked cellulose nano/micro-fibrils and their fast shape recovery property in water. J. Mater. Chem..

[186] Murphy S.V., Atala A. (2014). 3D bioprinting of tissues and organs. Nat. Biotechnol..

[187] Rutz A.L., Hyland K.E., Jakus A.E., Burghardt W.R., Shah R.N. (2015). A multimaterial bioink method for 3D printing tunable, cell-compatible hydrogels. Adv. Mater..

[188] Chung J.H.Y., Naficy S., Yue Z., Kapsa R., Quigley A., Moulton S.E., Wallace G.G. (2013). Bio-ink properties and printability for extrusion printing living cells. Biomater. Sci..

[189] Dutta S.D., Hexiu J., Patel D.K., Ganguly K., Lim K.T. (2021). 3D-printed bioactive and biodegradable hydrogel scaffolds of alginate/gelatin/cellulose nanocrystals for tissue engineering. Int. J. Biol. Macromol..

[190] Patel D.K., Dutta S.D., Shin W.C., Ganguly K., Lim K.T. (2021). Fabrication and characterization of 3D printable nanocellulose-based hydrogels for tissue engineering. RSC Adv..

[191] Shuai C., Yuan X., Yang W., Peng S., He C., Feng P., Qi F., Wang G. (2020). Cellulose nanocrystals as biobased nucleation agents in poly-L-lactide scaffold: crystallization behavior and mechanical properties. Polym. Test..

[192] Yin K., Divakar P., Wegst U.G.K. (2019). Plant-derived nanocellulose as structural and mechanical reinforcement of freeze-cast chitosan scaffolds for biomedical applications. Biomacromolecules.

[193] Athinarayanan J., Periasamy V.S., Alshatwi A.A. (2021). Fabrication of cellulose nanocrystal-decorated hydroxyapatite nanostructures using ultrasonication for biomedical applications. Biomass Convers. Biorefinery.

[194] Al-Sabah A., Burnell S.E.A., Simoes I.N., Jessop Z., Badiei N., Blain E., Whitaker I.S. (2019). Structural and mechanical characterization of crosslinked and sterilised nanocellulose-based hydrogels for cartilage tissue engineering. Carbohydr. Polym..

[195] Tummala G.K., Joffre T., Rojas R., Persson C., Mihranyan A. (2017). Strain-induced stiffening of nanocellulose-reinforced poly (vinyl alcohol) hydrogels mimicking collagenous soft tissues. Soft Matter.

[196] Tummala G.K., Joffre T., Lopes V.R., Liszka A., Buznyk O., Ferraz N., Persson C., Griffith M., Mihranyan A. (2016). Hyperelastic nanocellulose-reinforced hydrogel of high water content for ophthalmic applications. ACS Biomater. Sci. Eng..

[197] He X., Xiao Q., Lu C., Wang Y., Zhang X., Zhao J., Zhang W., Zhang X., Deng Y. (2014). Uniaxially aligned electrospun all-cellulose nanocomposite nano fibers reinforced with cellulose nanocrystals: scaffold for tissue engineering. Biomacromolecules.

[198] Huang A., Peng X., Geng L., Zhang L., Huang K., Chen B., Gu Z., Kuang T. (2018). Electrospun poly (butylene succinate)/cellulose nanocrystals bio-nanocomposite scaffolds for tissue engineering: preparation, characterization and in vitro evaluation. Polym. Test..

[199] Patel D.K., Dutta S.D., Hexiu J., Ganguly K., Lim K.T. (2020). Bioactive electrospun nanocomposite scaffolds of poly(lactic acid)/cellulose nanocrystals for bone tissue engineering. Int. J. Biol. Macromol..

[200] Sultan S., Mathew A.P. (2018). 3D printed scaffolds with gradient porosity based on a cellulose nanocrystal hydrogel. Nanoscale.

[201] Maturavongsadit P., Narayanan L.K., Chansoria P., Shirwaiker R., Benhabbour S.R. (2021). Cell-laden nanocellulose/chitosan-based bioinks for 3D bioprinting and enhanced osteogenic cell differentiation. ACS Appl. Bio Mater..

[202] Li V.C.F., Kuang X., Mulyadi A., Hamel C.M., Deng Y., Qi H.J. (2019). 3D printed cellulose nanocrystal composites through digital light processing. Cellulose.

[203] Gao W., Sun L., Zhang Z., Li Z. (2020). Cellulose nanocrystals reinforced gelatin/bioactive glass nanocomposite scaffolds for potential application in bone regeneration. J. Biomater. Sci. Polym. Ed..

[204] Chen X., Zhou R., Chen B., Chen J. (2016). Nanohydroxyapatite/cellulose nanocrystals/silk fibroin ternary scaffolds for rat calvarial defect regeneration. RSC Adv..

[205] Kumar A., Negi Y.S., Choudhary V., Bhardwaj N.K. (2016). Fabrication of poly (vinyl alcohol)/ovalbumin/cellulose nanocrystals/nanohydroxyapatite based biocomposite scaffolds. Int. J. Polym. Mater. Polym. Biomater..

[206] Naseri N., Deepa B., Mathew A.P., Oksman K., Girandon L. (2016). Nanocellulose-based Interpenetrating polymer network (IPN) hydrogels for cartilage applications. Biomacromolecules.

[207] Siqueira P., Siqueira É., de Lima A.E., Siqueira G., Pinzón-Garcia A.D., Lopes A.P., Segura M.E.C., Isaac A., Pereira F.V., Botaro V.R. (2019). Three-dimensional stable alginate-nanocellulose gels for biomedical applications: towards tunable mechanical properties and cell growing. Nanomaterials.

[208] Silva R.M., Pereira F.V., Mota F.A.P., Watanabe E., Soares S.M.C.S., Santos M.H. (2016). Dental glass ionomer cement reinforced by cellulose microfibers and cellulose nanocrystals. Mater. Sci. Eng. C..

[209] Hong J.K., Cooke S.L., Whittington A.R., Roman M. (2021). Bioactive cellulose nanocrystal-poly(ε-caprolactone) nanocomposites for bone tissue engineering applications. Front. Bioeng. Biotechnol..

[210] Pooyan P., Kim I.T., Jacob K.I., Tannenbaum R., Garmestani H. (2013). Design of a cellulose-based nanocomposite as a potential polymeric scaffold in tissue engineering. Polymer (Guildf).

[211] de Castro D.O., Bras J., Gandini A., Belgacem N. (2016). Surface grafting of cellulose nanocrystals with natural antimicrobial rosin mixture using a green process. Carbohydr. Polym..

[212] Feese E., Sadeghifar H., Gracz H.S., Argyropoulos D.S., Ghiladi R.A. (2011). Photobactericidal porphyrin-cellulose nanocrystals: synthesis, characterization, and antimicrobial propertiesorth States. United, Biomicromolecules..

[213] Tang J., Song Y., Tanvir S., Anderson W.A., Berry R.M., Tam K.C. (2015). Polyrhodanine coated cellulose nanocrystals: a sustainable antimicrobial agent. ACS Sustain. Chem. Eng..

[214] Peres B.U., Manso A.P., Carvalho L.D., Ko F., Troczynski T., Vidotti H.A., Carvalho R.M. (2019). Experimental composites of polyacrilonitrile-electrospun nanofibers containing nanocrystal cellulose. Dent. Mater..

[215] Zhang C., Salick M.R., Cordie T.M., Ellingham T., Dan Y., Turng L.S. (2015). Incorporation of poly(ethylene glycol) grafted cellulose nanocrystals in poly(lactic acid) electrospun nanocomposite fibers as potential scaffolds for bone tissue engineering. Mater. Sci. Eng. C..

[216] Hivechi A., Bahrami S.H., Siegel R.A. (2019). Drug release and biodegradability of electrospun cellulose nanocrystal reinforced polycaprolactone. Mater. Sci. Eng. C..

[217] Wang K., Nune K.C., Misra R.D.K. (2016). The functional response of alginate-gelatin-nanocrystalline cellulose injectable hydrogels toward delivery of cells and bioactive molecules. Acta Biomater..

[218] Ajdary R., Huan S., Zanjanizadeh Ezazi N., Xiang W., Grande R., Santos H.A., Rojas O.J. (2019). Acetylated nanocellulose for single-component bioinks and cell proliferation on 3D-printed scaffolds. Biomacromolecules.

[219] Xu C., Molino B.Z., Wang X., Cheng F., Xu W., Molino P., Bacher M., Su D., Rosenau T., Willfor S., Wallace G. (2018). 3D printing of nanocellulose hydrogel scaffolds with tunable mechanical strength towards wound healing application. J. Mater. Chem. B.

[220] Rasheed A., Azizi L., Turkki P., Janka M., Hytönen V.P., Tuukkanen S. (2021). Extrusion-based bioprinting of multilayered nanocellulose constructs for cell cultivation using in situ freezing and preprint CaCl2 cross-linking. ACS Omega.

[221] Deng Y., Xi J., Meng L., Lou Y., Seidi F., Wu W., Xiao H. (2022). Stimuli-Responsive nanocellulose Hydrogels: an overview. Eur. Polym. J..

[222] Subhedar A., Bhadauria S., Ahankari S., Kargarzadeh H. (2021). Nanocellulose in biomedical and biosensing applications: a review. Int. J. Biol. Macromol..

[223] Oprea M., Panaitescu D.M. (2020). Nanocellulose hybrids with metal oxides nanoparticles for biomedical applications. Molecules.

[224] Li W., Wang S., Li Y., Ma C., Huang Z., Wang C., Li J., Chen Z., Liu S. (2017). One-step hydrothermal synthesis of fluorescent nanocrystalline cellulose/carbon dot hydrogels. Carbohydr. Polym..

[225] Ferreira F.V., De Souza L.P., Martins T., Lopes J.H.H., Mattos B.D., Mariano M., Pinheiro I.F., Valverde T.M., Livi S., Camilli J.A., Goes A., Gouveia R.F., Lona L.F., Rojas O.J. (2019). Nanocellulose/bioactive glass cryogel as scaffolds for bone regeneration. Nanoscale.

[226] Abdi M.M., Razalli R.L., Tahir P.M., Chaibakhsh N., Hassani M., Mir M. (2019). Optimized fabrication of newly cholesterol biosensor based on nanocellulose. Int. J. Biol. Macromol..

[227] Li J., Cha R., Mou K., Zhao X., Long K., Luo H., Zhou F., Jiang X. (2018). Nanocellulose-based antibacterial materials. Adv. Healthc. Mater..

[228] Liu Z., Chen M., Guo Y., Zhou J., Shi Q., Sun R. (2020). Oxidized nanocellulose facilitates preparing photoluminescent nitrogen-doped fluorescent carbon dots for Fe3+ ions detection and bioimaging. Chem. Eng. J..

[229] Sun X., Tyagi P., Agate S., McCord M.G., Lucia L.A., Pal L. (2020). Highly tunable bioadhesion and optics of 3D printable PNIPAm/cellulose nanofibrils hydrogels. Carbohydr. Polym..

[230] Nguyen T.H.M., Abueva C., Van Ho H., Lee S.-Y., Lee B.-T. (2018). In vitro and in vivo acute response towards injectable thermosensitive chitosan/TEMPO-oxidized cellulose nanofiber hydrogel. Carbohydr. Polym..

[231] Kuzmenko V., Karabulut E., Pernevik E., Enoksson P., Gatenholm P. (2018). Tailor-made conductive inks from cellulose nanofibrils for 3D printing of neural guidelines. Carbohydr. Polym..

[232] Ajdary R., Ezazi N.Z., Correia A., Kemell M., Huan S., Ruskoaho H.J., Hirvonen J., Santos H.A., Rojas O.J. (2020). Multifunctional 3D-printed patches for long-term drug release therapies after myocardial infarction. Adv. Funct. Mater..

[233] Abouzeid R.E., Khiari R., Beneventi D., Dufresne A. (2018). Biomimetic mineralization of three-dimensional printed alginate/TEMPO-oxidized cellulose nanofibril scaffolds for bone tissue engineering. Biomacromolecules.

[234] Cheng H., Xiao D., Tang Y., Wang B., Feng X., Lu M., Vancso G.J., Sui X. (2020). Sponges with Janus character from nanocellulose: preparation and applications in the treatment of hemorrhagic wounds. Adv. Healthc. Mater..

[235] Martínez Ávila H., Schwarz S., Rotter N., Gatenholm P. (2016). 3D bioprinting of human chondrocyte-laden nanocellulose hydrogels for patient-specific auricular cartilage regeneration. Bioprinting.

[236] Möller T., Amoroso M., Hägg D., Brantsing C., Rotter N., Apelgren P., Lindahl A., Kölby L., Gatenholm P. (2017). In vivo chondrogenesis in 3D bioprinted human cell-laden hydrogel constructs. Plast. Reconstr. Surg. - Glob. Open..

[237] Chinga-Carrasco G., Ehman N.V., Filgueira D., Johansson J., Vallejos M.E., Felissia F.E., Håkansson J., Area M.C. (2019). Bagasse—a major agro-industrial residue as potential resource for nanocellulose inks for 3D printing of wound dressing devices. Addit. Manuf..

[238] Carlström I.E., Rashad A., Campodoni E., Sandri M., Syverud K., Bolstad A.I., Mustafa K. (2020). Cross-linked gelatin-nanocellulose scaffolds for bone tissue engineering. Mater. Lett..

[239] Tang A., Li J., Li J., Zhao S., Liu W., Liu T., Wang J., Liu Y. (2019). Nanocellulose/PEGDA aerogel scaffolds with tunable modulus prepared by stereolithography for three-dimensional cell culture. J. Biomater. Sci. Polym. Ed..

[240] Ghafari R., Jonoobi M., Amirabad L.M., Oksman K., Taheri A.R. (2019). Fabrication and characterization of novel bilayer scaffold from nanocellulose based aerogel for skin tissue engineering applications. Int. J. Biol. Macromol..

[241] Abudula T., Saeed U., Memic A., Gauthaman K., Hussain M.A., Al-Turaif H. (2019). Electrospun cellulose Nano fibril reinforced PLA/PBS composite scaffold for vascular tissue engineering. J. Polym. Res..

[242] Zhang X., Morits M., Jonkergouw C., Ora A., Valle-Delgado J.J., Farooq M., Ajdary R., Huan S., Linder M., Rojas O., Sipponen M.H., Österberg M. (2020). Three-dimensional printed cell culture model based on spherical colloidal lignin particles and cellulose nanofibril-alginate hydrogel. Biomacromolecules.

[243] De Chen R., Huang C.F., hui Hsu S. (2019). Composites of waterborne polyurethane and cellulose nanofibers for 3D printing and bioapplications. Carbohydr. Polym..

[244] Baniasadi H., Ajdary R., Trifol J., Rojas O.J., Sepp J. (2021). Direct ink writing of aloe vera/cellulose nanofibrils bio-hydrogels. Carbohydr. Polym..

[245] Mohan D., Khairullah N.F., How Y.P., Sajab M.S., Kaco H. (2020). 3D printed laminated CaCO3-nanocellulose films as controlled-release 5-fluorouracil. Polymers.

[246] Xu W., Molino B.Z., Cheng F., Molino P.J., Yue Z., Su D., Wang X., Willför S., Xu C., Wallace G.G. (2019). On low-concentration inks formulated by nanocellulose assisted with gelatin methacrylate (GelMA) for 3D printing toward wound healing application. ACS Appl. Mater. Interfaces.

[247] Fourmann O., Hausmann M.K., Neels A., Schubert M., Nyström G., Zimmermann T., Siqueira G. (2021). 3D printing of shape-morphing and antibacterial anisotropic nanocellulose hydrogels. Carbohydr. Polym..

[249] Rashad A., Suliman S., Mustafa M., Pedersen T., Campodoni E., Sandri M., Syverud K., Mustafa K. (2019). Inflammatory responses and tissue reactions to wood-Based nanocellulose scaffolds. Mater. Sci. Eng. C..

[250] Oberweis C.V., Marchal J.A., López-Ruiz E., Gálvez-Martín P. (2020). A Worldwide overview of regulatory frameworks for tissue-based products. Tissue Eng. Part B Rev..

[251] Lu L., Arbit H.M., Herrick J.L., Segovis S.G., Maran A., Yaszemski M.J. (2015). Tissue engineered constructs: perspectives on clinical translation. Ann. Biomed. Eng..

[252] Williams D.F. (2008). On the mechanisms of biocompatibility. Biomaterials.

[253] ISO 10993-1:2018: biological evaluation of medical devices — Part 1: evaluation and testing within a risk management process. https://www.iso.org/standard/68936.html.

[254] ISO 10993-12:2021: biological evaluation of medical devices — Part 12: sample preparation and reference materials. https://www.iso.org/standard/75769.html.

[255] ISO 10993-18:2020: biological evaluation of medical devices — Part 18: chemical characterization of medical device materials within a risk management process. https://www.iso.org/standard/64750.html.

[256] ISO/TS 10993-19:2020 Biological evaluation of medical devices — Part 19: Physico-chemical, morphological and topographical characterization of materials. https://www.iso.org/standard/75138.html.

[257] ISO 10993-17:2002 Biological evaluation of medical devices — Part 17: Establishment of allowable limits for leachable substances. https://www.iso.org/standard/23955.html.

[258] ISO/CD 10993-17.2 Biological evaluation of medical devices — Part 17: toxicological risk assessment of medical device constituents. https://www.iso.org/standard/75323.html.

[259] ISO 10993-4:2017 Biological evaluation of medical devices — Part 4: selection of tests for interactions with blood. https://www.iso.org/standard/63448.html.

[260] ISO 10993-3:2014 Biological evaluation of medical devices — Part 3: tests for genotoxicity, carcinogenicity and reproductive toxicity. https://www.iso.org/standard/55614.html.

[261] Peneda Pacheco D., Suárez Vargas N., Visentin S., Petrini P. (2021). From tissue engineering to engineering tissues: the role and application of in vitro models. Biomater. Sci..

[262] ISO/IEC 17025: General requirements for the competence of testing and calibration laboratories. https://www.iso.org/files/live/sites/isoorg/files/store/en/PUB100424.pdf.

[263] Association for assessment and accreditation of laboratory animal care (AAALAC). https://www.aaalac.org/.

[264] Database of privately and publicly funded clinical studies conducted around the world. https://clinicaltrials.gov/.

[265] Classification of medical device: FDA activities application and approvals. https://www.fda.gov/medical-devices/overview-device-regulation/classify-your-medical-device.

[266] Device advice: comprehensive regulatory assistance. https://www.fda.gov/medical-devices/device-advice-comprehensive-regulatory-assistance.

[267] Cfr - Code of federal regulations. https://www.accessdata.fda.gov/scripts/cdrh/cfdocs/cfcfr/CFRSearch.cfm?CFRPart=807&showFR=1&subpartNode=21:8.0.1.1.5.5.

[268] Device classification panels: application and approvals FDA activities. https://www.fda.gov/medical-devices/classify-your-medical-device/device-classification-panels.

[269] Devices - FDA. https://www.accessdata.fda.gov/scripts/cdrh/devicesatfda/index.cfm.

[270] Fortunato E., Gaspar D., Duarte P., Pereira L., Águas H., Vicente A., Dourado F., Gama M., Martins R. (2016).

[271] De assis C.A., Houtman C., Pgilips R., (Ted) Bilek E.M., Rojas O.J., Pal L., Peresin M.S., Jameel H., Gonzalez R. (2017). Conversion economics of forest biomaterials: risk and ifnancial analysis of CNC manufacturing Camilla. Biofuels, Bioprod. Biorefining.

[272] Chen L., Zhu J.Y., Baez C., Kitin P., Elder T. (2016). Highly thermal-stable and functional cellulose nanocrystals and nanofibrils produced using fully recyclable organic acids. Green Chem..

[273] Charreau H., Cavallo E., Foresti M.L. (2020). Patents involving nanocellulose: analysis of their evolution since 2010. Carbohydr. Polym..

[274] Nelson K. (2020). Advancing commercialization of nanocellulose : critical challenges workshop report.

[275] Crowther T.W., Glick H.B., Covey K.R., Bettigole C., Maynard D.S., Thomas S.M., Smith J.R., Hintler G., Duguid M.C., Amatulli G., Tuanmu M.N., Jetz W., Salas C., Stam C., Piotto D., Tavani R., Green S., Bruce G., Williams S.J., Wiser S.K., Huber M.O., Hengeveld G.M., Nabuurs G.J., Tikhonova E., Borchardt P., Li C.F., Powrie L.W., Fischer M., Hemp A., Homeier J., Cho P., Vibrans A.C., Umunay P.M., Piao S.L., Rowe C.W., Ashton M.S., Crane P.R., Bradford M.A. (2015). Mapping tree density at a global scale. Nature.

[276] Group N.P. Cellulose nanofiber manufacturing technology and application development, Nippon Pap. Gr. https://www.nipponpapergroup.com/english/research/organize/cnf.html.

[277] FibDex® - NATURAL WOUND HEALING UPM biomed. http://www.upmbiomedicals.com/for-clinical/fibdex.

[278] ASICS (2018).

[279] Li T., Chen C., Brozena A.H., Zhu J.Y., Xu L., Driemeier C., Dai J., Rojas O.J., Isogai A., Wågberg L., Hu L. (2021). Developing fibrillated cellulose as a sustainable technological material. Nature.

[280] Zeng Z., Wang C., Siqueira G., Han D., Huch A., Abdolhosseinzadeh S., Heier J., Nüesch F., (John) Zhang C., Nyström G. (2020). Nanocellulose‐MXene biomimetic aerogels with orientation‐tunable electromagnetic interference shielding performance. Adv. Sci..

[281] Zeng Z., Wu T., Han D., Ren Q., Siqueira G., Nyström G. (2020). Ultralight, flexible, and biomimetic nanocellulose/silver nanowire aerogels for electromagnetic interference shielding. ACS Nano.

[282] Culver H.R., Clegg J.R., Peppas N.A. (2017). Analyte-responsive hydrogels: intelligent materials for biosensing and drug delivery. Acc. Chem. Res..

[283] Ma H., Killaars A.R., DelRio F.W., Yang C., Anseth K.S. (2017). Myofibroblastic activation of valvular interstitial cells is modulated by spatial variations in matrix elasticity and its organization. Biomaterials.

[284] Ferreira F.V., Otoni C.G., Lopes J.H., de Souza L.P., Mei L.H.I., Lona L.M.F., Lozano K., Lobo A.O., Mattoso L.H.C. (2021). Ultrathin polymer fibers hybridized with bioactive ceramics: a review on fundamental pathways of electrospinning towards bone regeneration. Mater. Sci. Eng. C..

[285] dos Santos D.M., Correa D.S., Medeiros E.S., Oliveira J.E., Mattoso L.H.C. (2020). Advances in functional polymer nanofibers: from spinning fabrication techniques to recent biomedical applications. ACS Appl. Mater. Interfaces.

[286] Momeni F., Mehdi HassaniN S.M., Liu X., Ni J. (2017). A review of 4D printing. Mater. Des..

[287] Gauss C., Pickering K., Muthe L.P. (2021). The use of cellulose in bio-derived formulations for 3D/4D printing: a review. Compos. Part C Open Access.

[288] Siebert L., Luna‐Cerón E., García‐Rivera L.E., Oh J., Jang J., Rosas‐Gómez D.A., Pérez‐Gómez M.D., Maschkowitz G., Fickenscher H., Oceguera‐Cuevas D., Holguín‐León C.G., Byambaa B., Hussain M.A., Enciso‐Martínez E., Cho M., Lee Y., Sobahi N., Hasan A., Orgill D.P., Mishra Y.K., Adelung R., Lee E., Shin S.R. (2021). Light‐controlled growth factors release on tetrapodal ZnO‐incorporated 3D‐printed hydrogels for developing smart wound scaffold. Adv. Funct. Mater..

[289] Talebian S., Mehrali M., Taebnia N., Pennisi C.P., Kadumudi F.B., Foroughi J., Hasany M., Nikkhah M., Akbari M., Orive G., Dolatshahi‐Pirouz A. (2019). Self‐healing hydrogels: the next paradigm shift in tissue engineering?. Adv. Sci..

[290] Li L., Yan B., Yang J., Chen L., Zeng H. (2015). Novel mussel-inspired injectable self-healing hydrogel with anti-biofouling property. Adv. Mater..

[291] Lei Z., Wang Q., Sun S., Zhu W., Wu P. (2017). A bioinspired mineral hydrogel as a self-healable, mechanically adaptable ionic skin for highly sensitive pressure sensing. Adv. Mater..

[292] Vanderfleet O.M., Cranston E.D. (2020). Production routes to tailor the performance of cellulose nanocrystals. Nat. Rev. Mater..

